# Antiprotozoal Natural Products from Endophytic Fungi Associated with Cacao and Coffee

**DOI:** 10.3390/metabo14110575

**Published:** 2024-10-25

**Authors:** Cristopher A. Boya P., Candelario Rodriguez, Randy Mojica-Flores, Jean Carlo Urrutia, Víctor Cantilo-Diaz, Masiel Barrios-Jaén, Michelle G. Ng, Laura Pineda, Alejandro Llanes, Carmenza Spadafora, Luis C. Mejía, Marcelino Gutiérrez

**Affiliations:** 1 Centro de Biodiversidad y Descubrimiento de Drogas, Instituto de Investigaciones Científicas y Servicios de Alta Tecnología (INDICASAT AIP), Panamá 0843-01103, Panama; cboya@indicasat.org.pa (C.A.B.P.); crodriguez@coiba.org.pa (C.R.); rmojica@indicasat.org.pa (R.M.-F.); jeancpty@gmail.com (J.C.U.); victor.gem.04@gmail.com (V.C.-D.); mbarrios@indicasat.org.pa (M.B.-J.); lmejia@indicasat.org.pa (L.C.M.); 2 Estación Científica COIBA AIP, Ciudad del Saber, Panamá 0816-02852, Panama; 3 Centro de Biología Molecular y Celular de Enfermedades (CBCME), Instituto de Investigaciones Científicas y Servicios de Alta Tecnología (INDICASAT AIP), Panamá 0843-01103, Panama; michelle.ng.w@gmail.com (M.G.N.); lpineda@indicasat.org.pa (L.P.); allanes@indicasat.org.pa (A.L.); cspadafora@indicasat.org.pa (C.S.); 4 Smithsonian Tropical Research Institute, Ancón 0843-03092, Panama

**Keywords:** Chagas disease, leishmaniasis, endophytes, antiprotozoals, *Trypanosoma cruzi*, *Leishmania donovani*

## Abstract

Background: Collectively, leishmaniasis and Chagas disease cause approximately 8 million cases and more than 40,000 deaths annually, mostly in tropical and subtropical regions. The current drugs used to treat these diseases have limitations and many undesirable side effects; hence, new drugs with better clinical profiles are needed. Fungal endophytes associated with plants are known to produce a wide array of bioactive secondary metabolites, including antiprotozoal compounds. In this study, we analyzed endophytic fungal isolates associated with *Theobroma cacao* and *Coffea arabica* crop plants, which yielded extracts with antitrypanosomatid activity. Methods: Crude extracts were subjected to bioassay-guided isolation by HPLC, followed by spectrometric and spectroscopic analyses via mass spectrometry (MS) and nuclear magnetic resonance (NMR), Results: Compounds **1**–**9** were isolated and displayed novel antitrypanosomal and antileishmanial activities ranging from 0.92 to 32 μM. Tandem liquid chromatography–mass spectrometry (LC–MS) analysis of the organic extracts from different strains via the feature-based Global Natural Products Social (GNPS) molecular networking platform allowed us to dereplicate a series of metabolites (**10**–**23**) in the extracts. Molecular docking simulations of the active compounds, using the 3-mercaptopyruvate sulfurtransferase protein from *L. donovani* (Ld3MST) and the cruzipain enzyme from *T. cruzi* as putative molecular targets, allowed us to suggest possible mechanisms for the action of these compounds. Conclusions: The isolation of these antiprotozoal compounds confirms that crop plants like coffee and cacao harbor populations of endophytes with biomedical potential that confer added value to these crops.

## 1. Introduction

Tropical diseases affect millions of people worldwide, mainly in the poorest regions, and can be caused by bacteria, fungi, viruses, and parasites. Among the parasitic diseases, those caused by protozoans such as American trypanosomiasis and leishmaniasis have been disregarded by biotechnological industries [1]. Approximately twelve thousand deaths are reported annually for American trypanosomiasis, while millions of new cases per year are estimated for leishmaniasis [2]. These diseases affect mainly low-income populations; however, travelers also represent a significant source for the spread of these diseases [1,2].

The protozoan *Trypanosoma cruzi* is the causative agent of Chagas disease. This zoonosis is endemic to the American continent and affects more than seven million people in 21 countries from the USA to Chile [3,4]. Nifurtimox and benznidazole are the only drugs available for the treatment of Chagas disease, and the urgency of new treatments is exacerbated by drug resistance, toxicity, and the inability to treat chronic infections effectively [5,6].

Leishmaniasis is caused by more than 20 species of the genus *Leishmania*. This zoonotic disease is transmitted to humans through the bites of phlebotomine sandflies. Once the parasite enters a human in its promastigote form, it transforms into amastigotes that infect other cells [7,8]. The current treatment for leishmaniasis still depends on the use of antiquated first-line drugs such as pentavalent antimonies [9]. Other treatments used to treat *Leishmania* infections have been developed via drug repurposing, including the antifungal agent amphotericin B [10], miltefosine, which was initially used in cancer [11], and paromomycin, an aminoglycoside antibiotic [12,13].

In the search for new treatments, some protein targets of different metabolic pathways have been suggested for the trypanosomatid parasites *T. cruzi* and *L. donovani*, such as trypanothione reductase for the biosynthesis of trypanothione; tubulin and enzymes related to sterol biosynthesis; the glycolytic pathway; the biosynthesis de novo of pyrimidine nucleotides; the salvage of purines nucleotide synthesis; and, largely, the major protease termed cruzipain, which is a protein that has been detected in all stages of *T. cruzi* [14,15,16].

In *Leishmania* protozoans, intracellular redox homeostasis depends on the biosynthesis of trypanothione and glutathione, both of which heavily rely on cysteine bioavailability [17,18]. *Leishmania* protozoans lack the enzymes required for the absorption of sulfide from external sources via the sulfide reduction pathway, hence resorting to 3-mercaptopyruvate (3MP) as the major source of sulfide by the action of the enzyme 3-mercaptopyruvate sulfurtransferase (3MST) [19,20]. 3MST has been proposed as a potential drug target in *L. donovani* because of its importance in parasite survival.

Fungal endophytes biosynthesize a wealth of natural products with a wide array of biological activities [21,22,23]. These microorganisms colonize plant tissues without causing apparent damage and are known to contribute to the health and survival of plants [24]. Like most tropical plants surveyed, crop trees such as *Theobroma cacao* and *Coffea arabica* harbor a rich diversity of endophytic fungi that play key ecological roles in defending their host against pathogens such as *Moniliophthora roreri* (Frosty Pod Rot), *Phytophthora palmivora* (Black Pod Rot), *Moniliophthora perniciosa* (Witches’ Broom), *Hemileia vastatrix* (coffee leaf rust), and *Colletotrichum theobromicola* (Anthracnose) [25,26,27,28,29,30,31,32,33]. In addition to their ecological role, the natural products produced by fungal endophytes have proven to be important sources of antiparasitic compounds [21,22,23].

As part of our search for antiprotozoal compounds, we analyzed a series of endophytic fungal isolates from *T. cacao* and *C. arabica* from our institutional collection. This collection contains 3500 endophytic fungal isolates from *C. arabica* and 1000 from *T. cacao*. Biological screening of this collection has yielded extracts with activity against *Trypanosoma cruzi* and *Leishmania donovani* parasites. A bioassay-guided isolation of selected fungal extracts led to the isolation of compounds **1**–**9**, most of which presented novel biological activities against *T. cruzi* and *L. donovani* in the range of 0.92 to 32 μM. Furthermore, MS-based mining of these extracts via feature-based molecular networking and the GNPS platform allowed the identification of additional metabolites **10**–**23**. To gain a better understanding of the mechanism of action of these compounds, molecular docking studies with the enzyme cruzipain from *T. cruzi* and 3-mercaptopyruvate sulfurtransferase protein (Ld3MST) from *L. donovani* were carried out.

## 2. Materials and Methods

### 2.1. Isolation and Identification of Fungal Endophytes from Theobroma cacao

Endophytic fungi were isolated from the fruits of *T. cacao* collected in 2019 in the Province of Bocas del Toro, Panama. For the isolation of endophytes, the cacao fruit epicarp was processed via surface sterilization following the methods described by Mejía et al. [26]. Fungal isolates TCF400 and TCF417 were identified as *Waltergamsia zeylanica* and *Clonostachys rosea*, respectively, based on sequence analysis of the rDNA ITS region. DNA extraction, amplification of the ITS region, and sequencing of the two isolates were performed as previously described [26,34,35]. A sequence identity equal to or greater than 99% of the reference material in the National Center for Biological Information (NCBI) GenBank database served as a criterion for naming the isolates. The sequences were deposited in the GenBank database under the accession numbers PP732393 for *W. zeylanica* TCF400, and PQ270274 for *C. rosea* TCF417. The isolates were also deposited in the microbial biobank of the INDICASAT AIP as *W. zeylanica* TCF400 and *C. rosea* TCF417.

### 2.2. Isolation and Identification of Fungal Endophytes from Coffea arabica

Endophytic fungi were isolated from leaves of the *C. arabica* variety Geisha, collected in 2021 in the localities of Río Cristal and Cañas Verdes, Boquete District, in the highlands of the Province of Chiriquí, Panama. The isolates GEF-7-34.1 and GEF-7-28.5 were identified as *Multiguttulispora* sp. and *Xylaria grammica*, respectively, based on sequence analysis of the rDNA ITS region. DNA extraction, amplification of the ITS region, and sequencing were performed as previously described [34,35]. A sequence identity equal to or greater than 98% of the reference material in the NCBI GenBank database served as a criterion for naming the isolates to the genus rank. The sequences were deposited in the GenBank database under the accession numbers PP738328 for *Multiguttulispora* sp. GEF-7-34.1, and PP862734 for *X. grammica* GEF-7-28.5. The isolates were also deposited in the Microbial Biobank of the INDICASAT AIP as *Multiguttulispora* sp. GEF-7-34.1 and *Xylaria grammica* GEF-7-28.5.

### 2.3. General Experimental Procedures

NMR spectra were measured on a JEOL ECZR 500 MHz spectrometer (Jeol, Peabody, MA, USA). Chemical shifts were referenced internally to the residual signals of deuterated chloroform (CDCl_3_ δ_H_ 7.26, δ_C_ 77.16), acetone (acetone-*d*6 δ_H_ 2.05, δ_C_ 29.84), methanol (CD_3_OD δ_H_ 3.32, δ_C_ 49.0), or pyridine (C_5_D_5_N δ_H_ 7.19, 7.55 δ_C_ 123.5). For the NMR measurements, 0.2–5 mg of the compound (depending on the amount available) was dissolved in 225 µL of deuterated solvent. HPLC separations were performed via an Agilent 1260 Infinity II system equipped with a quaternary pump, a diode array detector (Agilent, Santa Clara, CA, USA), and reverse-phase columns (Phenomenex Synergi Hydro-RP, 10 × 250 mm, 5 µm; Kinetex^®^ EVO, 10 × 250 mm, 5 µm; and Synergi Polar-RP, 10 × 250 mm, 4 µm; Phenomenex, Torrance, CA, USA). Solid-phase extraction (SPE) separation was carried out via SUPELCO Supelclean™ LC-C18 (octadecyl) solid-phase extraction cartridges (Supelco^®^ Analytical, Bellefonte, PA, USA).

### 2.4. Extraction and Isolation of Secondary Metabolites from Theobroma cacao Endophytes

Fungal strains *W. zeylanica* and *C. rosea* were cultivated separately on 200 plates of malt extract agar and incubated for 31 days at room temperature. Each of the fungal cultures was sonicated, followed by maceration with 1200 mL of ethyl acetate (EtOAc) of ACS grade for 24 h with constant agitation. The organic extracts were filtered through a 1.0 μm fiberglass filter and dried under reduced pressure to yield 1.29 g of *C. rosea* extract and 0.80 g of *W. zeylanica* extract.

The crude EtOAc extract of *C. rosea* was fractionated using open-column chromatography employing silica gel as the stationary phase. Elution was carried out starting with hexanes followed by a gradient system of 20, 40, 60, 80, and 100% EtOAc in hexane, resulting in six fractions (CF1–CF6). The purification of the active compounds was guided by bioactivity assays against *L. donovani* and *T. cruzi* parasites. The active fraction CF5 (80% EtOAc) was dissolved in methanol (1 mg/20 µL) and purified via high-performance liquid chromatography (HPLC) using a semipreparative C_18_ reverse-phase column (Synergi Hydro-RP 10 mm × 250 mm), which was eluted with a gradient system of 70–80% methanol in water for 25 min, followed by isocratic elution with 100% methanol for 35 min, resulting in the isolation of compound **1** (4.4 mg).

The EtOAc extract of *W. zeylanica* was fractionated in a C-18 SPE cartridge (10 g) and eluted in a stepped gradient with 100 mL of 20%, 40%, 60%, 80%, and 100% methanol in water, yielding five fractions (WF1-WF5). Fractions were assessed via biological assays against *L. donovani* and *T. cruzi* parasites, and the antitrypanosomal and antileishmanial activities were detected in fraction WF4 (80% methanol). This fraction was further purified via high-performance liquid chromatography (HPLC) using a semipreparative C_18_ column (Kinetex^®^ EVO 10 mm × 250 mm) eluted with a gradient system from 80% methanol in water, increased to 87.5% methanol in water for 40 min, and returned to 80% methanol in water in 5 min at a flow rate of 2 mL/min, resulting in 15 fractions. Fraction WF4-9 was further purified via an isocratic elution system of 80% acetonitrile in water at a flow rate of 1.5 mL/min, leading to the isolation of compounds **2** (3.7 mg), **3** (0.9 mg), and **4** (0.2 mg), which were subjected to complete characterization analysis via LC–MS and NMR.

### 2.5. Extraction and Isolation of Secondary Metabolites from Coffea arabica Endophytes

Isolates of *Multiguttulispora* sp. and *Xylaria grammica* were cultivated separately in potato dextrose agar media for 15 days at room temperature. Each of the fungal cultures was sonicated for 5 min in an ultrasonic bath, followed by maceration with 200 mL of EtOAc of ACS grade overnight with constant agitation at 115 R.P.M. The organic extracts were filtered through a 1.0 μm fiberglass filter without a binder. The remaining mycelia were washed three times with 200 mL of EtOAc and filtered, and the filtrates were pooled and concentrated in vacuo to yield 110.1 mg of *Multiguttulispora* sp. extract and 1516.1 mg of *X. grammica* extract. The EtOAc extract from *Multiguttulispora* sp. was loaded into the SPE cartridge (10 g) and eluted sequentially with 100 mL of 20%, 40%, 60%, 80%, or 100% methanol in water, yielding five fractions (MF1-MF5). The same procedure was used to obtain five fractions of *X. grammica*, XF1-XF5. Antileishmanial and antitrypanosomal bioassays revealed activity in fraction MF2 from the *Multiguttulispora* sp. extract and fractions XF1 and XF2 from the *X. grammica* extract. 

Hence, the active fraction MF2 was further purified via reverse-phase HPLC using a semipreparative C18 column (Kinetex^®^ EVO 10 mm × 250 mm) eluted with a gradient of 40% to 52% methanol in water for 30 min, followed by an increase to 60% methanol in water for 40 min and a return to 40% methanol in water for 45 min at a flow rate of 2 mL/min to yield 14 fractions. NMR analysis revealed that fractions MF2-8 (0.3 mg) and MF2-9 (4.0 mg) contained pure compounds **5** and **6**, respectively. Similarly, the active SPE fraction XF1 (85.0 mg) from *X. grammica* was purified via reverse-phase HPLC using a semipreparative C18 column (Synergy Polar-RP 10 mm × 250 mm), eluted with 35% to 80% methanol in water for 35 min, increased to 100% methanol for 50 min, stayed at 100% methanol for 70 min, and returned to 35% methanol in water for 80 min at a flow rate of 1 mL/min to yield 19 fractions. NMR data revealed that fractions XF1-1 (0.5 mg), XF1-3 (53.8 mg), and XF1-6 (0.6 mg) contained pure compounds **7**–**9**, respectively.

### 2.6. Liquid Chromatography–Mass Spectrometry Analyses

The crude extracts were fractionated via SPE C-18 cartridges and eluted with a stepped gradient of 10 mL of 20%, 40%, 60%, 80%, 100% methanol in water, and dichloromethane methanol (50%:50%), yielding six fractions. Each fraction was resuspended in methanol at a concentration of 0.5 mg/mL and analyzed via an ACQUITY UPLC chromatographic system (Waters Corporation, Milford, MA, USA) equipped with a Kinetex^®^ 1.7 µm C18 100 Å, LC 50 × 2.1 mm column (Phenomenex Inc., Torrance, CA, USA) coupled to a Xevo TQD triple quadrupole mass spectrometer (Waters Corporation, Milford, MA, USA) with an electrospray ionization (ESI) source. Chromatographic separation was carried out for 25 min using a gradient of methanol (A) and acidified water (99.9% water and 0.1% formic acid) (B), a 14 min gradient from 10% A and 90% B to 100% A. then held at 100% B for 8 min and returned to initial conditions, with a flow rate of 0.3 mL/min throughout the process. MS spectra were acquired via positive ESI ionization via survey data collection mode, with the mass range for MS 150–2000 *m*/*z* and the range for fragmentation (MS/MS) 50–2000 *m*/*z*. The selected collision energies were 20 eV and 30 eV, and the activation intensity was adjusted to 1 × 105. To switch between MS and MS/MS modes, the selected ions for fragmentation were excluded for 30 s after 3 consecutive scans.

### 2.7. GNPS Feature-Based Molecular Networking (FBMN)

The spectroscopic data obtained (.raw) were converted to the universal .mzXML file format via the subset unit at MSconvert [36]. First, the subset of MS1 scans was corrected and merged with MS2 data via MZmine 3.9.0 [37,38]. MZmine processing was subsequently performed as follows: peaks were detected with a noise factor of 15 for MS1 and 0 for MS2; scan-to-scan accuracy was set to 0.25 *m*/*z* for building chromatograms; chromatograms were resolved via the local minimum algorithm with a 95% chromatographic threshold and peak duration of 0.2–2 min; isotopic peaks were grouped and then aligned with an RT tolerance of 0.2 min and the same accuracy; and blank features were filtered out; the resulting .mgf and quantification table were exported to feature-based molecular networking (FBMN) [37,38].

Feature-based molecular networking (FBMN) was processed via the GNPS (Global Natural Product Social Molecular Networking) computational platform [39,40,41]. A molecular network was created with a precursor ion mass tolerance of 0.25 Da and an MS/MS fragment ion tolerance of 0.75 Da. Correlations were filtered to have a cosine score above 0.6 and more than 3 matching peaks. The spectra in the network were then searched against the GNPS spectral libraries. Matching peaks were manually curated and, for each dereplicated compound, we generated a direct comparison of fragmentation patterns over the Metabolomics Spectrum Resolver and the Universal Spectrum Identifier (USI) [42].

### 2.8. Bioassays

#### 2.8.1. Antitrypanosomal Activity

Antitrypanosomal bioassays were performed using the recombinant strain Tulahuen, clone C4, of *T. cruzi* (ATCC, Manassas, VA, USA) in the intracellular form. This strain expresses the enzyme β-galactosidase when viable [43]. The parasites were incubated at 37 °C in an atmosphere of 5% CO_2_ in RPMI-1640 culture medium supplemented with L-glutamine, 4-(2-hydroxyethyl)-piperazine-1-ethanesulfonic acid (HEPES) buffer, 3.2% NaHCO_3_ (*v*/*w*), 10% FBS, and 0.05% gentamicin (50 mg/mL). Epithelial monkey kidney Vero cells (ATCC, Manassas, VA, USA) were incubated for 24 h and then infected with parasites 24 h prior to the addition of the experimental compounds. After an additional 24 h of infection, compounds **1**–**9** were dissolved in DMSO and tested at 10, 2, 0.4, and 0.08 µg/mL for five days. The antichagasic drug benznidazole served as the positive control. To quantify the biological activity, chlorophenol-red-β-D-galactopyranoside (Roche Applied Science, Penzberg, Germany) was added to each well, and the mixture was then allowed to react with the parasite β-galactosidase from living parasites for 4.5 h. The absorbance was measured at 570 nm via a plate reader (Sinergy HT, BioTek Instruments Inc., Winooski, VT, USA). 

#### 2.8.2. Antileishmanial Activity

The samples and parasites were incubated for 72 h. The number of parasites in the culture was determined via the DNA cross-linking agent PicoGreen^®^ solution (1%), which was added to the wells, and the plates were incubated in the dark. The plates were read in a microplate reader set to 485/20 nm for the excitation step and to 528/20 nm for the emission step. The positive control was amphotericin B for the *L. donovani* assay [44].

#### 2.8.3. Cytotoxicity Assay

Epithelial cells from monkey kidney (Vero) cells were incubated at 37 °C in 96-well plates under an atmosphere of 5% CO_2_ in RPMI-1640 medium (Sigma-Aldrich, St. Louis, MO, USA) supplemented with 0.05% gentamicin (50 mg/mL) and 10% FBS (fetal bovine serum; Gibco, Invitrogen, Carlsbad, CA, USA). The cells were allowed to adhere for one day before being incubated for three days with the test compounds. Doxorubicin and culture media were used as positive and negative controls, respectively. When the incubation period ended, 3-(4,5-di-methyl-thiazol-2-yl)-2,5-di-phenyl-tetra-zolium bromide (MTT) was added to the wells and, after 4 h, the absorbance was measured at two wavelengths, 570 nm and 630 nm, via a color plate reader. Cytotoxicity was assessed colorimetrically by calculating the capacity of the remaining living Vero cells to reduce yellow MTT to the dark purple formazan product, as previously reported [45].

#### 2.8.4. Statistical Analysis of the Bioassays

All bioassays represent independent analyses and were carried out in duplicate. The data analysis complement Wizard of Excel 2000 (Microsoft, Seattle, WA, USA) was used for the statistical analysis of the 50% inhibitory concentration (IC_50_) and cytotoxic concentration (CC_50_) values by adjusting the dose–response curve to a sigmoidal model.

### 2.9. Molecular Docking Analysis

The crystallographic structures of protein targets were retrieved from the Protein Data Bank (PDB) database [46]. Exploratory molecular docking simulations were selected with all chosen protein targets, whereas a refined selection of docking binding sites was later performed for the targets with the best preliminary docking results for each parasite, namely, the *L. major* 3-mercaptopyruvate sulfurtransferase (3MST) [47] for *Leishmania* and cruzipain for *T. cruzi* [48]. All structures were manually cleaned by removing non-biologically active components, such as solvents, ions, dimers, prosthetic groups, and anisotropy lines from the original PDB file. The missing electronic density for each structure was built with Swiss-Model, and the three-dimensional (3D) homology model of the unresolved structure for *L. donovani* 3MST (*Ld*3MST) was built following the methodology described by Vijayakumar et al. [49] with Modeller 10.5 [50], using the amino acid sequence retrieved from the protein database in the NCBI (accession number XP_003858284.1), with the *L. major* 3MST (*Lm*3MST) as a template (PDB ID 1OKG), 3-mercaptopyruvate as substrate, and a cocrystallized sulfide ion (Figures S28 and S29). The quality of this model was assessed with ERRAT, VERIFY3D, and Ramachandran plots integrated into the Protein Structure Analysis and Verification Server (SAVES) v6.0 (https://saves.mbi.ucla.edu/, accessed on September 6, 2024) [51,52,53]. The July 2022 port of the CHARMM36 all-atom protein forcefield was employed for the final correction of the distance, angles, and torsion over the final structures via energy minimization with the steepest descent protocol integrated in GROMACS v2022.3 [54,55].

The structures of active compounds **1**–**4**, **6**, and **8** were retrieved from the PubChem database in the isomeric version of the simplified molecular-input line-entry system (SMILES) format [56]. Each one-dimensional structure was converted to two-dimensional depictions with the AllChem module integrated with the RDKit cheminformatics package [57]. The experimental torsion knowledge distance geometry (ETKDGv3) method [58] was employed for embedding with explicit hydrogens the 3D structures of the compounds by the generation of one thousand conformers, and the optimization of their geometry was performed with the standard Merk Molecular Mechanics Force Field (MMFF94) [59].

File conversion tasks were completed via OpenBabel v3.1.0 [60], and final refinements of the structures were performed via the semiempirical quantum mechanical methods Geometry, Frequency, Noncovalent, and eXtended Tight-Binding 2 (GFN2-xTB) [61], a geometry optimization method with greater accuracy than traditional forcefields. The preparation of the protein–ligand complexes and the subsequent generation of atom maps were carried out with AutoDockTools v1.5.7 [62]. Molecular docking simulations were performed with AutoDock-GPU v1.5 in batch mode via the gradient-based search method ADADELTA [63]. The centers of the coordinates for the grid box were chosen as the positions occupied by the catalytic residues Cys25 and His162 for cruzipain, residue Cys253 for *Ld*3MST, and the center of mass of the cocrystallized inhibitors or substrates of the remaining targets, each grid box with a radius of 10 Å. For comparative purposes, the final conformers were overlaid with the noncovalent inhibitors or the natural substrates cocrystallized with each target’s structure [64].

## 3. Results and Discussion

### 3.1. Isolation and Identification of Metabolites from Theobroma cacao Endophytes

**Figure 1:**
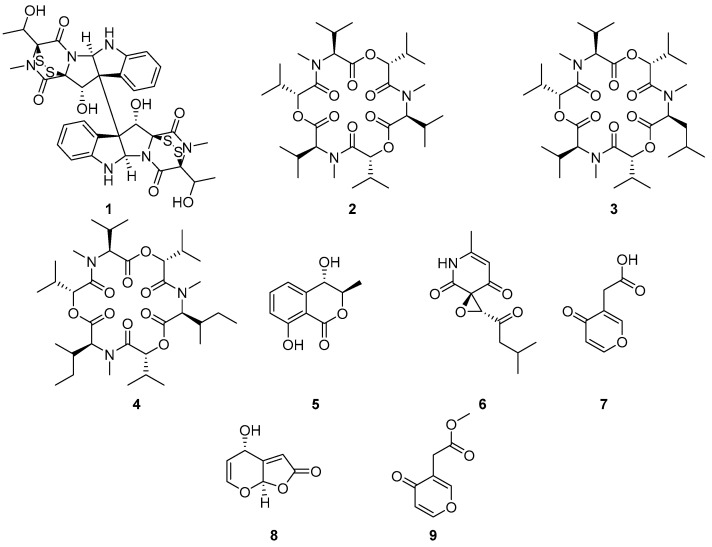
Metabolites isolated from *T. cacao* (**1**–**4**) and *C. arabica* (**5**–**9**) endophytic fungi.

The crude extract from *C. rosea* was fractionated using silica gel column chromatography to yield six fractions, and fraction CF5 (80% EtOAc) was active in the *L. donovani* assay. Further HPLC purification of CF5 led to the isolation of compound **1** (4.4 mg) (Figure 1), which was identified as verticillin D after detailed NMR and MS analyses and comparisons with spectroscopic data from the literature [65].

Similarly, the crude extract from *W. zeylanica* was fractionated via SPE C-18 cartridges to obtain five fractions, and fraction WF4 (80% methanol) was active in the *T. cruzi* and *L. donovani* bioassays; hence, it was further purified via HPLC, yielding compounds **2** (3.7 mg), **3** (0.9 mg), and **4** (0.2 mg). Compounds **2**–**4** were identified as enniatin B (**2**) [66], enniatin B4 (**3**) [67,68], and enniatin A1 (**4**) [69,70,71] after NMR and MS/MS analyses and comparisons with spectroscopic data from the literature.

### 3.2. Isolation and Identification of Metabolites from Coffea arabica Endophytes

Strains of *Multiguttulispora* sp. and *Xylaria grammica* were cultivated on potato dextrose agar. Agars containing the endophytic fungi were extracted with EtOAc and fractionated using SPE C-18, yielding five fractions (F1–F5) per extract. The SPE fractions were subjected to antileishmanial and antitrypanosomal bioassays, and the active fractions were further purified via reverse-phase high-performance liquid chromatography (HPLC) to yield compounds **5**–**6** from *Multiguttulispora* sp. and **7**–**9** from *X. grammica* (Figure 1). Compounds **5**–**6** were identified as trans-3,4-hydroxymellein (**5**) [72] and flavipucine (**6**) [73], whereas compounds **7**–**9** were identified as xylaric acid (**7**) [74], grammicin (**8**) [75], and the methyl ester of xylaric acid (**9**) [75] after NMR analyses and comparisons with spectroscopic data from the literature.

### 3.3. Feature-Based Molecular Networking

Using feature-based molecular networking, we further explored the chemical diversity of crop-associated endophytes. The fractionated extracts were analyzed via ultrahigh-performance liquid chromatography coupled with mass spectrometry (UHPLC-MS). Data analysis was performed via feature-based molecular networking on the GNPS (Global Natural Product Social Molecular Networking) computational platform of the University of California San Diego [39]. As a result, 14 additional compounds were dereplicated from the endophytes.

**Figure 2:**
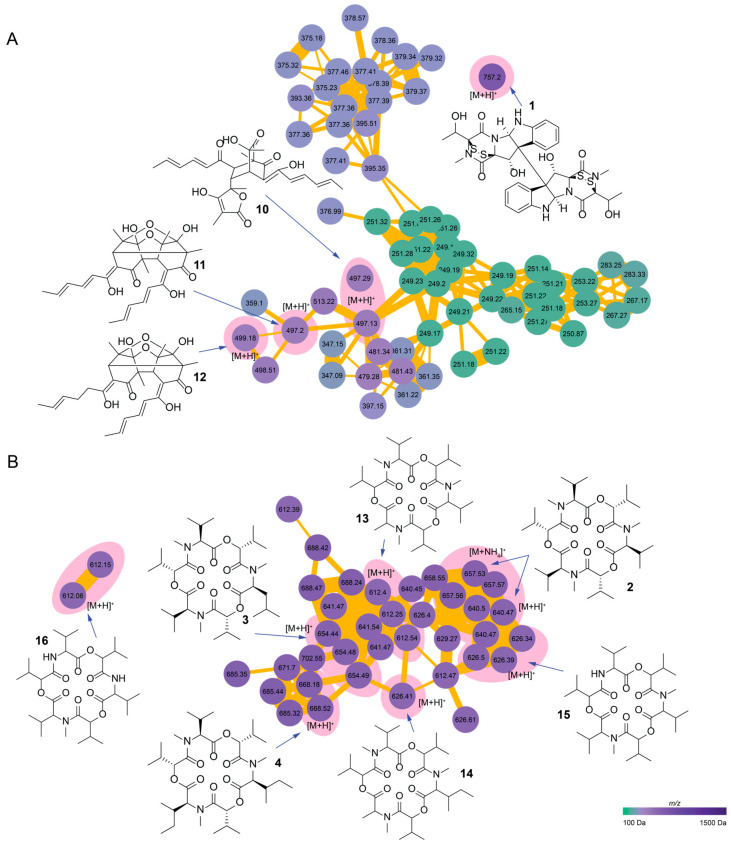
GNPS feature-based molecular networks of *T. cacao-*associated endophytes. (**A**) *C. rosea* network, sorbicillinoid molecular cluster, and verticillin D. (**B**) *W. zeylanica* network, cyclodepsipeptide molecular cluster highlighting the enniatin family. The pink shadow denotes dereplicated compounds and adducts. The node color represents the *m*/*z* gradient of each feature detected.

Fractions obtained from the *T. cacao*-associated endophyte *C. rosea* network presented 1247 features as nodes and 1792 edges (Figure S1), of which 676 were self-loops and 781 were connected features. Connected features were gathered in different-sized clusters, and curation of the network led to the identification of compound **1** (Figure 2) as a singleton showing the [M+H]^+^ adduct with a mass of 757.20 Da (Figure S5).

Manual annotation resulted in the dereplication of the sorbicillinoid molecular cluster. Sorbicillinoids are hexaketide metabolites featuring intricate, highly oxygenated bicyclic and tricyclic structures [76]. We were able to dereplicate bislongiquinolide (**10**) [77], trichodimerol (**11**) [78], and dihydrotrichodimerol (**12**) [79] (Figure 2A) by direct comparison of fragmentation patterns through the Metabolomics Spectrum Resolver and the Universal Spectrum Identifier (USI) [42]. For each compound, we generated a mirror plot of the spectrum and the database standard (Figures S6–S8). Additionally, we manually corroborated the fragmentation pattern. Compounds **11** and **12** have been previously isolated from other strains of *C. rosea* [80,81], and compound **10** was reported tentatively via UPLC MS [82].

*W. zeylanica* fractions were analyzed via the same approach; as a result, we obtained a network comprising 785 features (Figure S2). The isolated enniatin compounds **2**–**4** clustered into 38 nodes that were highly interconnected with 121 edges (Figure 2B) (Figures S9–S11). Dereplication at GNPS and curation allowed the identification of three additional known enniatins: enniatin J1 (**13**), enniatin J2 (**14**), and enniatin B2 (**15**) (Figures S12–S14). Manual curation and fragmentation analysis of the ion at *m*/*z* 612.06 led to the identification of enniatin B3 (**16**) on a small cluster of two nodes (Figure S15). Our manual MS/MS fragmentation analysis of the enniatin derivatives revealed distinct product ions and fragment losses that validated each identification, as previously reported [69,70,71].

The FBMN chemical analysis of *C. arabica*-associated endophytes revealed chemotypes closely related to the compounds (**5**–**6**) isolated from *Multiguttulispora* sp. and, in the case of *X. grammica*, we identified compounds that had previously been isolated from the genus *Xylaria* [83,84].

The molecular network analysis of *Multiguttulispora* sp. revealed a structure comprising 759 features as nodes (Figure S3), which were interconnected through 1115 edges and featured 488 self-loop nodes. Inconspicuously, the compound (**5**) was found as a self-loop node; therefore, the nearby cluster included the related compound mellein (**17**) [85], which is a metabolic precursor of compound **5**. Additionally, fusaric acid (**18**) [86] was identified within the same cluster in its [M+H]^+^ form, further supporting the interconnectedness of these metabolites (Figure S18).

The primary cluster highlights the [M+H]^+^ adduct of flavipucine (**6**), which is part of a molecular family that consists of 65 nodes. Annotation of these clusters led to the identification of several related compounds, including isoflavipucine (**19**) [87], dihydroisoflavipucine (**20**) [73,87], and the tautomeric methoxyl acetal of isoflavipucine, previously cataloged as MoNA:VF-NPL-QEHF019532 (**21**) [88,89] (Figures S20–S22). This intricate network also underscores several nodes we could not dereplicate, indicating the biochemical diversity and a potential source of novel compounds for further exploration.

**Figure 3:**
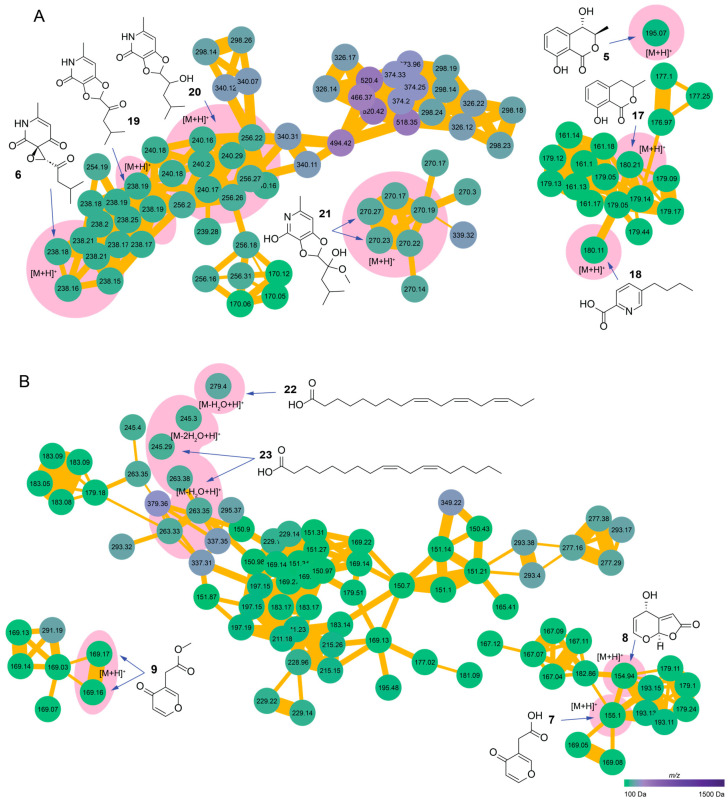
GNPS feature-based molecular networks of *C. arabica-*associated endophytes. (**A**) *Multiguttulispora* sp. network and pyridone polyketide molecular cluster highlighting the flavipucine family, as well as 4-hydroxymellein, mellein, and fusaric acid. (**B**) *X. grammica* network, polyunsaturated fatty acid molecular cluster, and grammicin analogs. The pink shadow denotes dereplicated compounds and adducts. The node color represents the *m*/*z* gradient of each feature detected.

In the case of *X. grammica*, the analysis yielded a molecular network composed of 972 features (Figure 3B and Figure S4). This network confirmed the presence of xylaric acid (**7**), grammicin (**8**), and the methyl ester of xylaric acid (**9**), which were identified in the form of [M+H]^+^ adducts with masses of 154.94 Da, 155.08 Da, and 168.99 Da, respectively.

Furthermore, linoleic acid (**22**) [83] and linolenic acid (**23**) [84] were successfully dereplicated from the extract of this fungus based on spectral comparisons (Figures S26 and S27).

These findings on crop-associated endophytes not only enhance our understanding of the chemical ecology of these fungi but also provide a groundwork for forthcoming research into their potential applications as building blocks for subsequent chemical modifications to be used in pharmacology.

### 3.4. Antiprotozoal Activity

Bioassay-guided fractionation of extracts obtained from the crop-associated endophytic fungi *C. rosea*, *W. zeylanica*, *Multiguttulispora* sp., and *X. grammica* led to the isolation of a variety of natural products (**1**–**9**) belonging to different structural classes, including epipolythiodioxopiperazines, depsipeptides, polyketides, and coumarines. Compounds **1**–**9** were evaluated for their activity against *Leishmania donovani* and *Trypanosoma cruzi* parasites and were shown to have novel antiprotozoal activity (Table 1).

Compounds **1**, **2,** and **6** showed low micromolar antileishmanial activity, with verticillin D (**1**) being the most potent, with an effective concentration (EC50) value of 0.92 µM, which is close to that of the standard amphotericin B (Table 1). However, verticillin D (**1**) also resulted in the highest toxicity in the Vero cell assay. On the other hand, enniatin B (**2**) and flavipucine (**6**) showed better cytotoxic profiles and a better selectivity index in the *Leishmania* assay (Table 1).

Furthermore, compounds **1**–**9** were evaluated for their activity against *T. cruzi* parasites. In this assay, enniatins **2**–**4** showed low micromolar activity of the same order of magnitude as the standard drug benznidazole, with enniatin A1 (**4**) being less cytotoxic (36.14 μM) and hence having a better selectivity index of 5.15. Flavipucin (**6**) also showed moderate activity against *T. cruzi.*

Despite verticillin D (**1**), enniatins (**2**–**4**), and flavipucine (**6**) showing good antiprotozoal profiles, their cytotoxicity might raise concerns regarding their potential use as drugs [90]. The use of enniatins in anticancer therapy, as well as further preclinical studies, has been suggested due to their potent activity toward proliferating malignant cells [91]. In the case of enniatin A1 (**4**), compared with those of enniatins B and B4 (**2**–**3**), the presence of two isoleucine residues substantially improved the cytotoxicity to Vero cells (36.14 μM) and thus the selectivity index (5.15) (Table 1). These results warrant continuing work with this scaffold to improve its activity and selectivity via the synthesis of analogs.

The modern development of drug delivery systems (DDSs), such as antibody–drug conjugates (ADCs), has effectively overcome developmental problems encountered by anticancer [92,93] and antimicrobial drugs [93] from several natural products that were stagnant for many years or decades due to toxicity issues and that are currently commercial drugs. These ADCs are composed of a bioactive natural product (usually with toxicity problems) as a payload, that is coupled to a monoclonal antibody through a linker. ADCs are delivered directly to the receptors of cancer cells and release bioactive substances inside the cells, avoiding toxicity to normal cells.

Similarly, another DDS is composed of metal nanoparticles that have been effectively used to deliver natural products to macrophages containing *Leishmania* parasites. A review recently published by Ronconi et al. gives a significant number of examples of this approach [94]. However, no metal nanoparticle formulations to treat leishmaniasis have yet reached the market, and only one formulation is in clinical trials [94], very likely because of the lack of research on this illness; this might change in the future, as these DDSs are the focus of many new research programs, and many payloads, such as verticillins and enniatins, will be needed to support this research.

### 3.5. Molecular Docking Simulations

Our results reveal that crop-associated endophytic fungi are important sources of metabolites with antiprotozoal properties, such as compounds **1**–**4**, **6**, and **8**. In an attempt to propose a possible mechanism for the antitrypanosomal and antileishmanial activity of this compounds, we performed preliminary molecular docking simulations with protein targets commonly used in studies involving the respective parasites (Figure S30). Although possibly favorable interactions were observed in some of the selected targets, we focused on those with the best preliminary predictions for the vast majority of our compounds. For this selection, we considered targets exhibiting reasonably good binding scores, with docking poses similar to those of cocrystallized substrate/inhibitors, while also mimicking the molecular interactions previously reported with key amino acids in the binding sites. These targets were cruzipain for compounds with antitrypanosomal activity (compounds **2**–**4** and **6**) and 3-mercaptopyruvate sulfurtransferase (3MST) for those with antileishmanial activity (compounds **1**–**2**, **6**, and **8**).

Cruzipain is the major cysteine protease in *T. cruzi* and is part of the papain family of proteolytic enzymes. Various investigations have reported that its inhibition interrupts the differentiation and replication of the parasite, also affecting its nutritional cycle. Cruzipain is essential for multiple physiological processes and is expressed in all stages of the parasite life cycle, making it a potential drug target against *T. cruzi* [15]. In *Leishmania*, 3MST is a protein involved in protection mechanisms against oxidative stress, which are vital for parasite survival. *Leishmania* protozoans rely on thiol-containing antioxidants such as trypanothione and glutathione, whose biosynthesis depends on cysteine availability [17,18]. The de novo cysteine biosynthesis pathway, which is absent in humans, involves a bienzyme complex requiring sulfide [19,95,96,97]. *Leishmania* parasites, which lack enzymes for sulfide absorption, utilize 3-mercaptopyruvate (3MP) as a sulfide source through the action of 3MST [19,20]. 3MST is upregulated under sulfur and peroxide stress and is present in both the promastigote and amastigote stages, with low sequence homology to human enzymes [49].

**Figure 4:**
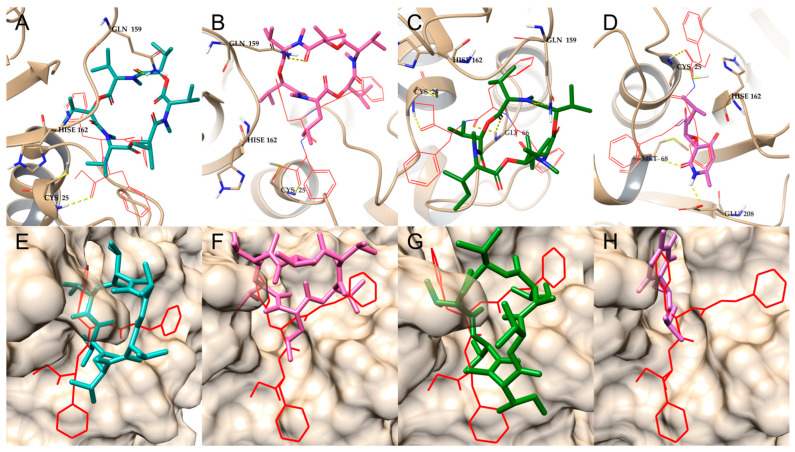
Molecular docking simulations of the interactions of isolated compounds **2**–**4** and **6** with cruzipain. Compounds **2** (**A**,**E**), **3** (**B**,**F**), **4** (**C**,**G**), and **6** (**D**,**H**) interact with key amino acids of cruzipain. H-bond interactions are depicted as dashed yellow lines at distances lower than 3.0 Å. Only polar hydrogens are shown for clarity. The compounds are displayed in stick representation compared with the orientation of the cocrystallized hydroxy methyl ketone noncovalent inhibitor, depicted with solid red lines.

Molecular docking simulations of antitrypanosomal compounds **2**–**4** and **6** were performed via AutoDock-GPU within the active site of cruzipain (Figure 4). As shown in Table 2, compounds **2**–**4** and **6** display similar binding scores (−5.63 to −6.32 kcal/mol) to those obtained after redocking the cocrystallized noncovalent inhibitor hydroxymethyl ketone (HMK) (−7.96 kcal/mol). Enniatin B4 (**3**) scored slightly higher than compounds **2**, **4**, and **6** did, which is consistent with the quantitative measures of antitrypanosomal activity shown in Table 1.

Compounds **2**–**4** are in close proximity to the catalytic Cys25, potentially anchoring hydrogen bond (H-bond) interactions with the side chain of Gln159 (Figure 4A–C), also suggesting stabilization by hydrophobic interactions in a pocket formed by residues Trp26, Gly65, Gly66, Leu67, and Leu160. Although the simulations also suggest a similar orientation for compound **6** in the same hydrophobic pocket, as well as its interaction via H-bonding with residues Met68 and Glu208 (Figure 4D), the smaller size of this compound may result in a lower stability within the solvent-exposed cavity of the active site, possibly contributing to its moderate antitrypanosomal activity. Moreover, a comparison of the docking poses predicted for compounds **2**–**4** and **6** revealed that several moieties of these compounds overlap with those of the cocrystallized HMK inhibitor, such as the peptide bonds or the phenyl aromatic ring (Figure 4E–H).

**Figure 5:**
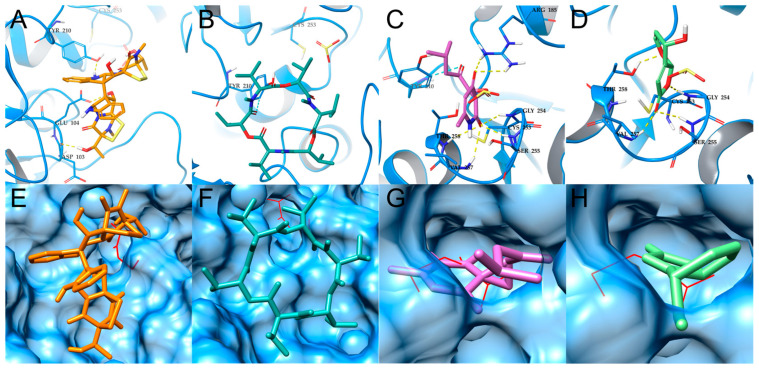
Molecular docking interactions of isolated compounds **1**–**2**, **6**, and **8** with Ld3MST. Compounds **1** (**A**,**E**), **2** (**B**,**F**), **6** (**C**,**G**), and **8** (**D**,**H**) interact with critical amino acids of Ld3MST. H-bond and pi–pi stacking interactions are depicted as dashed yellow and blue lines, respectively, at distances lower than 3.0 Å. Only polar hydrogens are shown for clarity. The isolated compounds are displayed in stick representation, in contrast to the orientation of the natural substrate of Ld3MST, 3MP, depicted with red lines, and to the superposition of the sulfite ions cocrystallized in the active site of the Lm3MST template.

A similar approach was used for the molecular docking simulations of antileishmanial compounds **1**, **2**, **6**, and **8** within the active site of the previously verified *Ld*3MST model (Figure 5). Compound **6** was predicted to fully occupy the active site of *Ld*3MST, with a docking score of −5.02 kcal/mol (Table 2). The lowest-energy docking suggested the possible formation of several stabilizing interactions via H-bonds through the carbonyl moieties in the pyridione ring, potentially anchoring compound **6** with a series of amino groups in the backbone of residues Gly254, Ser255, Val257, and Thr258, and the guanidine side chain of Arg185. The orientation of compound **6** in the active site may block access to the catalytic Cys253 residue at the bottom of the active site, thus potentially affecting the catalytic activity of *Ld*3MST (Figure 5C).

Similarly, compound **8** was also predicted to occupy the cavity of the *Ld*3MST active site (Figure 5H), with a docking score of −5.45 kcal/mol. This compound was predicted to interact through carbonyl H-bonds with the amino groups in the backbone of residues Gly254, Ser255, and Val257, and with the hydroxyl side chain of Thr258, potentially blocking access to the catalytic residue Cys253 (Figure 5D). The docking poses of compounds **6** and **8** overlap with the docked position of 3-MP and with the sulfite ion cocrystallized with *Ld*3MST in the original structure of the template (Figure 5G,H), which was previously reported to act as an inhibitor in similar rhodanese enzymes [49,98,99].

Compounds **1** and **2** possess a bulky structure that may affect their access to the active site of Ld3MST (Figure 5E,F), but potentially stabilizing H-bonds were still predicted with amino acid residues from the active site of this enzyme. However, our preliminary molecular docking simulations suggested that these compounds could interact more effectively with enzymes with larger and more open active sites such as pteridine reductase 1 (PTR1), for which both compounds exhibited some degree of overlapping with the cocrystallized inhibitor methotrexate (Figure S30).

Although further studies are required to characterize and validate the preliminary suggestions about the molecular mechanism for the actions of the compounds discussed in this study, these findings highlight the relevance of analyzing, via computational simulations, the plausible interactions of our bioactive compounds presenting in vitro antitrypanosomal and antileishmanial activity, further exploring the latent potential that remains hidden in the crop-associated microbiome.

## 4. Conclusions

In this study, we evaluated extracts from fungal isolates associated with *T. cacao* and *C. arabica* crop plants for their antitrypanosomatid activity. The bioassay-guided fractionation of active extracts led to the isolation of a variety of natural products (**1**–**9**) belonging to different structural classes, including epipolythiodioxopiperazines, depsipeptides, polyketides, and coumarines. Compounds **1**–**9** showed novel antiprotozoal activities against *L. donovani* and *T. cruzi*. Although verticillin D (**1**), enniatins (**2**–**3**), and flavipucin (**6**) showed good antiprotozoal profiles, they were also cytotoxic in the Vero cell assay. On the other hand, enniatin A1 (**4**) possessed a better cytotoxicity profile and selectivity index, suggesting that chemical modifications of this structural class could improve the selectivity index, creating better candidates for drug discovery. Feature-based molecular networking analyses revealed the presence of additional compounds (**10**–**23**). Furthermore, the low micromolar activity detected in vitro for molecules predicted in silico to affect target proteins in the two parasites attests to the validity of the method presented here.

## Figures and Tables

**Table 1:** Antiprotozoal activity and mammalian cytotoxicity of metabolites isolated from crop-associated endophytic fungi.

Compounds	*T. cruzi*	*L. donovani*	Vero Cell	Selectivity	Fungi/Host
	EC50	EC50	CC50	*Tc*/*Ld*	
**1**	I	0.92	0.31	0.34	*C. rosea*/*Theobroma cacao*
**2**	6.25	7.41	8.64	1.38/1.16	*W. zeylanica*/*Theobroma cacao*
**3**	5.74	I	10.4	1.81	
**4**	7.01	I	36.14	5.15	
**5**	I	I	-	-	*Multiguttulispora* sp./*Coffea arabica*
**6**	24.19	5.39	6.83	0.28/1.27	
**7**	I	I	-	-	*X. grammica*/*Coffea arabica*
**8**	>100	32.12	141.54	4.41	
**9**	I	I	-	-	
Amphotericin B	-	0.139	-		
Benznidazole	3.46	-	-		
Doxorubicin	-	-	1.21		

The effective (EC50) and cytotoxic (CC50) concentrations are presented as the mean values in µM. I: inactive.

**Table 2:** Key interactions and binding scores predicted by molecular docking simulations for the compounds from crop-associated endophytic fungi evaluated in this study.

Protein Target	Compounds	Interacting Amino Acid Residues (≤3.0 Å)		Binding Score (kcal/mol)
		Hydrogen Bonds and Polar Interactions	Hydrophobic Interactions (Van der Waals)	
Cruzain (*T. cruzi*)	Enniatin B (**2**)	**Gln159 ^a^**, Asp161	Gly23, Trp26, Gly65, Gly66, Leu67, Ala138, Leu160	−5.63
	Enniatin B4 (**3**)	Ser61, **Gln159 ^a^**, Asp161, His162	Trp26, Gly65, Gly66, Leu67, Ala138, Leu160	−6.32
	Enniatin A1 (**4**)	Ser64, **Gly66 ^a^**, **Gln159 ^a^**, Asp161, His162	Gly23, Trp26, Gly65, Leu67, Leu160	−5.91
	Flavipucin (**6**)	**Met68 ^a^**, Asp161, His162, **Glu208 ^a^**	Cys25, Trp26, Gly66, Leu67, Ala138, Leu160	−5.81
	Hydroxy methyl ketone ^b^	**Gln19 ^a^**, **Cys25 ^a^**, Asp60, **Gly66 ^a^**, Asp161, His162	Gly23, Trp26, Ser61, Gly65, Leu67, Ala138, Leu160	−7.96
Ld3MST (*L. donovani*)	Verticillin D (**1**)	Ser36, **Asp103 ^a^**, **Glu104 ^a^**, **Tyr210 ^a^,** Arg185	Leu37, Gln132, Arg185, Thr211, Leu214, Lys221	−5.41
	Enniatin B (**2**)	Ser36, Lys38, Asp103, Glu104, Thr211, Lys221	Leu37, Met108, Tyr210 ^c^, Leu214	−6.61
	Flavipucin (**6**)	Arg74, His75, Arg181, **Arg185 ^a^**, **Gly254 ^a^**, **Ser255 ^a^**, **Val257 ^a^**, **Thr258 ^a^**	Leu37, Met60, Tyr210 ^c^, Cys253	−5.02
	Grammicin (**8**)	Arg185, **Gly254 ^a^**, **Ser255 ^a^**, **Val257 ^a^**, **Thr258 ^a^**	Leu37, Tyr210, Cys253	−5.45
	3-MP ^d^	**Arg74 ^a^**, **Arg185 ^a^**, **Ser255 ^a^**, **Val257 ^a^**, **Thr258 ^a^.**	Leu37, Cys253, Gly254	−5.66

^a^ Residues highlighted in bold are those involved in interactions through hydrogen bonds. ^b^ Noncovalent inhibitor cocrystallized in the structure of cruzipain used as a template for docking, included here for comparative purposes. ^c^ Pi–Pi stacking interactions. ^d^ Natural substrate for the *Ld*3MST enzyme, included here for comparative purposes.

## Data Availability

Molecular networking results and parameters can be found online: for *C. rosea* at https://gnps.ucsd.edu/ProteoSAFe/status.jsp?task=6c61325e8f2149d18878d633b177aea5 (accessed on 3 September 2024); *W. zeylanica* at https://gnps.ucsd.edu/ProteoSAFe/status.jsp?task=15933a4303e64a1f95c62ac009b78727 (accessed on 3 September 2024); *Multiguttulispora* sp. at https://gnps.ucsd.edu/ProteoSAFe/status.jsp?task=5d0520f4d1e242a696af59d44ce139ea (accessed on 1 September 2024); *X. grammica* at https://gnps.ucsd.edu/ProteoSAFe/status.jsp?task=36cd1a54558f4d79911a79292d2e08e5 (accessed on 1 September 2024); NMR data are available upon request from the corresponding author.

## Supplementary Materials

The following supporting information can be downloaded at: https://www.mdpi.com/article/10.3390/metabo14110575/s1. Figure S1: GNPS Feature-based Molecular Networks of *T. cacao* associated endophytes *C. rosea*. Figure S2: GNPS Feature-based Molecular Networks of *T. cacao* associated endophytes *W. zeylanica*. Figure S3: GNPS Feature-based Molecular Networks of *C. arabica* associated endophytes *Multiguttulispora* sp. Figure S4: GNPS Feature-based Molecular Networks of *C. arabica* associated endophytes *X. grammica*. Figure S5: Metabolomics spectrum resolver mirror plot of verticillin D (**1**) and GNPS Library Spectrum CCMSLIB00000478454 at the Universal Spectrum Identifier (USI). Figure S6: Metabolomics spectrum resolver mirror plot of bislongiquinolide (**10**) and GNPS Library Spectrum CCMSLIB00004712865 at the Universal Spectrum Identifier (USI). Figure S7: Metabolomics spectrum resolver mirror plot of trichodimerol (**11**) and GNPS Library Spectrum CCMSLIB00000855732 at the Universal Spectrum Identifier (USI). Figure S8: Metabolomics spectrum resolver mirror plot of dihydrotrichodimerol (**12**) and GNPS Library Spectrum CCMSLIB00000851924 at the Universal Spectrum Identifier (USI). Figure S9: Metabolomics spectrum resolver mirror plot of enniatin B (**2**) and GNPS Library Spectrum CCMSLIB00005727731 at the Universal Spectrum Identifier (USI). Figure S10: Metabolomics spectrum resolver mirror plot of enniatin B4 (**3**) and GNPS Library Spectrum CCMSLIB00000577644 at the Universal Spectrum Identifier (USI). Figure S11: Metabolomics spectrum resolver mirror plot of enniatin A1 (**4**) and GNPS Library Spectrum CCMSLIB00005727903 at the Universal Spectrum Identifier (USI). Figure S12: Metabolomics spectrum resolver mirror plot of enniatin J1 (**13**) and GNPS Library Spectrum CCMSLIB00000577703 at the Universal Spectrum Identifier (USI). Figure S13: Metabolomics spectrum resolver mirror plot of enniatin J2 (**14**) and GNPS Library Spectrum CCMSLIB00000577620 at the Universal Spectrum Identifier (USI). Figure S14: Metabolomics spectrum resolver mirror plot of enniatin B2 (**15**) and GNPS Library Spectrum CCMSLIB00000577662 at the Universal Spectrum Identifier (USI). Figure S15: Metabolomics spectrum resolver mirror plot of enniatin B3 (**16**) and GNPS Library Spectrum CCMSLIB00000577824 at the Universal Spectrum Identifier (USI). Figure S16: Metabolomics spectrum resolver mirror plot of trans-3,4-hydroxymellein (**5**) and GNPS Library Spectrum CCMSLIB00000478502 (4-hydroxymellein) at the Universal Spectrum Identifier (USI). Figure S17: Metabolomics spectrum resolver mirror plot of mellein (**17**) and GNPS Library Spectrum CCMSLIB00005727561 at the Universal Spectrum Identifier (USI). Figure S18: Metabolomics spectrum resolver mirror plot of fusaric acid (**18**) and GNPS Library Spectrum CCMSLIB00005743636 at the Universal Spectrum Identifier (USI). Figure S19: Metabolomics spectrum resolver mirror plot of flavipucine (**6**) and GNPS Library Spectrum CCMSLIB00012725756 at the Universal Spectrum Identifier (USI). Figure S20: Metabolomics spectrum resolver mirror plot of isoflavipucine (**19**) and peak table reported by Findlay et al. 1977 as mgf format, uploaded to GNPS and imported to the Universal Spectrum Identifier (USI). Figure S21: Metabolomics spectrum resolver mirror plot of dihydroisoflavipucine (**20**) and GNPS Library Spectrum CCMSLIB00000854979 at the Universal Spectrum Identifier (USI). Figure S22: Metabolomics spectrum resolver mirror plot of the tautomeric methoxyl acetal of isoflavipucine (**21**) and GNPS Library Spectrum CCMSLIB00004710594 at the Universal Spectrum Identifier (USI). Figure S23: Metabolomics spectrum resolver mirror plot of xylaric acid (**7**) and GNPS Library Spectrum CCMSLIB00012474990 at the Universal Spectrum Identifier (USI). Figure S24: Metabolomics spectrum resolver mirror plot of grammicin (**8**) and GNPS Library Spectrum CCMSLIB00012474133 at the Universal Spectrum Identifier (USI). Figure S25: Metabolomics spectrum resolver mirror plot of the methyl ester of xylaric acid (**9**) and GNPS Library Spectrum CCMSLIB00012474993 at the Universal Spectrum Identifier (USI). Figure S26: Metabolomics spectrum resolver mirror plot of linoleic acid (**22**) and GNPS Library Spectrum CCMSLIB00011428768 at the Universal Spectrum Identifier (USI). Figure S27: Metabolomics spectrum resolver mirror plot of linolenic acid (**23**) and GNPS Library Spectrum CCMSLIB00005738688 at the Universal Spectrum Identifier (USI). Figure S28: Homology Modeling of the enzyme Ld3MST with the template Lm3MST (1OKG). Figure S29: Molecular docking pose of 3-mercaptopyruvate (3-MP). Figure S30: Preliminary molecular docking simulations performed with the active compounds and commonly used targets from *T. cruzi* and *Lesihmania*. References [65,66,67,68,69,70,71,72,73,75] are cited in the supplementary materials.
