# Supplementary material for: Antiprotozoal Natural Products from Endophytic Fungi Associated with Cacao and Coffee
Source: Metabolites. 2024 Oct 25;14(11):575. doi: 10.3390/metabo14110575 (PMC11596112; doi:10.3390/metabo14110575)
Supplement: Supplementary file 1 [file metabolites-14-00575-s001.zip › metabolites-3243304-supplementary.pdf]

metabolites

MDPI

Supporting Information

Antiprotozoal natural products from cacao and coffee associated endophytic fungi

Cristopher A. Boya P. [1,+], Candelario Rodriguez [1,2,+], Randy Mojica-Flores [1], Jean Carlos Urrutia [1,3], Víctor Cantilo-Diaz [1], Masiel Barrios Jaén [1], Michelle G. Ng [3], Alejandro Llanes [3], Carmenza Spadafora [3], Luis C. Mejía[1,4] and Marcelino Gutiérrez [1,*]

[1]Centro de Biodiversidad y Descubrimiento de Drogas - Instituto de Investigaciones Científicas y Servicios de Alta Tecnología (INDICASAT AIP), Panamá, Apartado 0843-01103, República de Panamá.
[2]Estación Científica COIBA AIP, Ciudad del Saber, Apartado 0816-02852, Panamá.
[3]Centro de Biología Molecular y Celular de Enfermedades (CBCME) - Instituto de Investigaciones Científicas y Servicios de Alta Tecnología (INDICASAT AIP), Panamá, Apartado 0843-01103, República de Panamá.
[4]Smithsonian Tropical Research Institute, Ancón 0843-03092, Panamá
*Correspondence: mgutierrez@indicasat.org.pa
[+]These authors contributed equally to this work

Contents

Contents 1

Spectroscopic data of the compounds.. 4

Figure S1. GNPS Feature-based Molecular Networks of *T. cacao* associated endophytes *C. rosea*. 5

Figure S2. GNPS Feature-based Molecular Networks of *T. cacao* associated endophytes *W. zeylanica*. 6

Figure S3. GNPS Feature-based Molecular Networks of *C. arabica* associated endophytes *Multiguttulispora sp.* 7

Figure S4. GNPS Feature-based Molecular Networks of *C. arabica* associated endophytes *X. grammica*. 8

Figure S5. Metabolomics spectrum resolver mirror plot of verticillin D (1) and GNPS Library Spectrum CCMSLIB00000478454 at the Universal Spectrum Identifier (USI). 9

Figure S6. Metabolomics spectrum resolver mirror plot of bislongiquinolide (10) and GNPS Library Spectrum CCMSLIB00004712865 at the Universal Spectrum Identifier (USI). 10

Figure S7. Metabolomics spectrum resolver mirror plot of trichodimerol (11) and GNPS Library Spectrum CCMSLIB00000855732 at the Universal Spectrum Identifier (USI). 11

Figure S8. Metabolomics spectrum resolver mirror plot of dihydrotrichodimerol (12) and GNPS Library Spectrum CCMSLIB00000851924 at the Universal Spectrum Identifier (USI). 12

Figure S9. Metabolomics spectrum resolver mirror plot of enniatin B (2) and GNPS Library Spectrum CCMSLIB00005727731 at the Universal Spectrum Identifier (USI). 13

Figure S10. Metabolomics spectrum resolver mirror plot of enniatin B4 (3) and GNPS Library Spectrum CCMSLIB00000577644 at the Universal Spectrum Identifier (USI). 14

Figure S11. Metabolomics spectrum resolver mirror plot of enniatin A1 (4) and GNPS Library Spectrum CCMSLIB00005727903 at the Universal Spectrum Identifier (USI). 15

Figure S12. Metabolomics spectrum resolver mirror plot of enniatin J1 (13) and GNPS Library Spectrum CCMSLIB00000577703 at the Universal Spectrum Identifier (USI). 16

Figure S13. Metabolomics spectrum resolver mirror plot of enniatin J2 (14) and GNPS Library Spectrum CCMSLIB00000577620 at the Universal Spectrum Identifier (USI). 17

Figure S14. Metabolomics spectrum resolver mirror plot of enniatin B2 (15) and GNPS Library Spectrum CCMSLIB00000577662 at the Universal Spectrum Identifier (USI). 18

Figure S15. Metabolomics spectrum resolver mirror plot of enniatin B3 (16) and GNPS Library Spectrum CCMSLIB00000577824 at the Universal Spectrum Identifier (USI). 19

Figure S16. Metabolomics spectrum resolver mirror plot of trans-3,4-hydroxymellein (5) and GNPS Library Spectrum CCMSLIB00000478502 (4-hydroxymellein) at the Universal Spectrum Identifier (USI)........ 20

Figure S17. Metabolomics spectrum resolver mirror plot of mellein (17) and GNPS Library Spectrum CCMSLIB00005727561 at the Universal Spectrum Identifier (USI). 21

Figure S18. Metabolomics spectrum resolver mirror plot of fusaric acid (18) and GNPS Library Spectrum CCMSLIB00005743636 at the Universal Spectrum Identifier (USI). 22

Figure S19. Metabolomics spectrum resolver mirror plot of flavipucine (6) and GNPS Library Spectrum CCMSLIB00012725756 at the Universal Spectrum Identifier (USI). 23

Figure S20. Metabolomics spectrum resolver mirror plot of isoflavipucine (19) and peak table reported by Findlay et al 1977 as mgf format, uploaded to GNPS and imported to the Universal Spectrum Identifier (USI)........ 24

Figure S21. Metabolomics spectrum resolver mirror plot of dihydroisoflavipucine (20) and GNPS Library Spectrum CCMSLIB00000854979 at the Universal Spectrum Identifier (USI)........ 25

Figure S22. Metabolomics spectrum resolver mirror plot of the tautomeric methoxyl acetal of isoflavipucine (21) and GNPS Library Spectrum CCMSLIB00004710594 at the Universal Spectrum Identifier (USI). 26

Figure S23. Metabolomics spectrum resolver mirror plot of xylaric acid (7) and GNPS Library Spectrum CCMSLIB00012474990 at the Universal Spectrum Identifier (USI). 27

Figure S24. Metabolomics spectrum resolver mirror plot of grammicin (8) and GNPS Library Spectrum CCMSLIB00012474133 at the Universal Spectrum Identifier (USI). 28

Figure S25. Metabolomics spectrum resolver mirror plot of the methyl ester of xylaric acid (9) and GNPS Library Spectrum CCMSLIB00012474993 at the Universal Spectrum Identifier (USI)........ 29

Figure S26. Metabolomics spectrum resolver mirror plot of linoleic acid (22) and GNPS Library Spectrum CCMSLIB00011428768 at the Universal Spectrum Identifier (USI). 30

Figure S27. Metabolomics spectrum resolver mirror plot of linolenic acid (23) and GNPS Library Spectrum CCMSLIB00005738688 at the Universal Spectrum Identifier (USI). 31

Figure S28. Homology Modeling of the enzyme *Ld*3MST with the template *Lm*3MST (1OKG). 32

Figure S29. Molecular docking pose of 3-mercaptopyruvate (3-MP). 33

Figure S30. Preliminary molecular docking simulations performed with the active compounds and commonly used targets from *T. cruzi* and *Lesihmania*........ 34

Reference 35

Spectroscopic data of the compounds

Verticillin D (**1**) [1]: White amorphous solid ^{1}H NMR (500 MHz, Pyridine-*D*$_5$) δ 8.35 (d, *J* = 7.7 Hz, 2H), 8.33 (s), 7.48 (s, 2H), 7.10 (t, *J* = 7.6 Hz, 2H), 6.74 (td, *J* = 7.5, 1.1 Hz, 2H), 6.68 (d, *J* = 7.8 Hz, 2H), 6.40 (s, 2H), 5.91 (s, 2H), 5.14 – 5.10 (m, 2H), 3.48 (s, 6H), 1.54 (d, *J* = 6.5 Hz, 6H); ^{13}C NMR (126 MHz, Pyridine-*D*$_5$) δ 167.74, 162.46, 151.21, 131.24, 130.33, 128.99, 119.76, 110.52, 83.84, 83.13, 82.25, 78.85, 67.85, 67.53, 29.87, 20.36; ESIMS *m*/*z*: [M + H]$^+$ 757.20

Enniatin B (**2**) [2]: White amorphous solid ^{1}H NMR (500 MHz, $CDCl_3$) δ 5.10 (d, *J* = 8.1 Hz, 1H), 4.57 (d, *J* = 9.7 Hz, 1H), 3.16 (s, 3H), 2.33 – 2.26 (m), 2.25 – 2.20 (m, 1H), 1.07 (d, *J* = 6.4 Hz, 3H), 0.99 (d, *J* = 6.5 Hz, 3H), 0.95 (d, *J* = 6.6 Hz, 3H), 0.92 (d, *J* = 6.6 Hz, 3H); ^{13}C NMR (126 MHz, $CDCl_3$) δ 170.707, 170.226, 75.841, 63.131, 33.035, 30.257, 28.140, 20.443, 19.931, 18.833, 18.644. ESIMS *m*/*z*: [M + H]$^+$ 640.66

Enniatin B4 (**3**) [3,4]: White amorphous solid ^{1}H NMR (500 MHz, $CDCl_3$) δ 5.16 (d, *J* = 8.3 Hz, 1H), 4.99 (d, *J* = 8.8 Hz, 2H), 4.91 (d, *J* = 10.0 Hz, 1H), 4.83 (s, 1H), 4.53 (d, *J* = 10.1 Hz, 1H), 3.18 (s, 3H), 3.15 (s, 3H), 3.14 (s, 3H), 2.28 – 2.18 (m, 6H), 1.87 – 1.78 (m, 1H), 1.76 – 1.67 (m, 1H), 1.55 – 1.47 (m, 1H), 1.05 (dd, *J* = 8.5, 6.6 Hz, 6H), 1.02 – 0.97 (m, 9H), 0.96 – 0.85 (m, 21H); ^{13}C NMR (125.76 MHz, $CDCl_3$) δ 171.05, 171.04, 170.92, 170.79, 170.75, 170.73, 75.58, 75.55, 75.53, 63.02, 61.77, 58.85, 55.86, 37.66, 32.62, 32.08, 30.77, 30.18, 30.14, 29.85, 28.17, 28.16, 25.51, 23.48, 20.25, 20.06, 19.08, 19.07, 18.89, 18.58, 18.56, 18.46, 18.29, 18.28; ESIMS *m*/*z*: [M + H]$^+$ 654.78

Enniatin A1 (**4**) [5–7]: White amorphous solid ^{1}H NMR (500 MHz, $CDCl_3$) δ 5.30 – 5.16 (m, 2H), 5.07 (d, *J* = 5.5 Hz, 1H), 4.99 (d, *J* = 8.7 Hz, 1H), 4.92 – 4.81 (m, 2H), 3.23 (s, 3H), 3.14 (d, *J* = 11.4 Hz, 6H), 2.35 (t, *J* = 7.5 Hz, 1H), 2.25 – 2.18 (m, 3H), 2.13 – 1.99 (m, 2H), 1.54 – 1.45 (m, 4H), 1.15 – 1.05 (m, 6H), 1.04 – 1.01 (m, 6H), 0.98 – 0.93 (m, 18H), 0.89 (d, *J* = 6.4 Hz, 6H); ESIMS *m*/*z*: [M + H]$^+$ 668.72

Trans-3,4-hydroxymellein (**5**) [8]: White amorphous solid; ^{1}H NMR (500 MHz, $CDCl_3$): δ 11.00 (s, 1H), 7.59 – 7.52 (m, 1H), 7.06 – 7.02 (m, 1H), 7.01 (dd, *J* = 8.4, 1.0 Hz, 1H), 4.63 (s, 1H), 4.68 – 4.56 (m, 2H), 1.53 (d, *J* = 6.1 Hz, 3H); ^{13}C NMR (125.76 MHz, $CDCl_3$) δ 168.58, 162.19, 141.26, 137.05, 118.02, 116.31, 106.80, 80.05, 69.33, 18.07; ESIMS *m*/*z*: [M + H]$^+$ 195.09.

Flavipucine (**6**) [9]: White crystalline solid; ^{1}H NMR (500 MHz, $CDCl_3$): ^{1}H NMR (500 MHz, CHLOROFORM-*D*) δ 8.87 (s, 1H), 5.64 (s, 1H), 3.84 (s, 1H), 2.73 (dd, *J* = 16.8, 6.4 Hz, 1H), 2.64 (dd, *J* = 16.8, 7.3 Hz, 1H), 2.18 (s, 3H), 2.17 (dp, *J* = 13.4, 6.7 Hz, 1H), 0.98 (d, *J* = 6.6 Hz, 3H), 0.94 (d, *J* = 6.7 Hz, 3H)); ^{13}C NMR (125.76 MHz, $CDCl_3$) δ 203.29, 186.26, 168.35, 155.30, 107.20, 68.75, 59.79, 49.57, 24.03, 22.91, 22.55, 20.90; ESIMS *m*/*z*: [M + H]$^+$ 238.24.

Xylaric acid (**7**) [8]: White amorphous solid; ^{1}H NMR (500 MHz, CD_3OD) δ 8.12 (s, 1H), 8.07 (d, *J* = 5.7 Hz, 1H), 6.40 (d, *J* = 5.7 Hz, 1H), 3.36 (s, 2H).; ^{13}C NMR (125.76 MHz, CD_3OD) δ 180.24, 174.09, 158.59, 156.90, 125.80, 117.04, 31.62; ESIMS *m*/*z*: [M + Na]$^+$ 177.11.

Grammicin (**8**) [10]: White amorphous solid; ^{1}H NMR (500 MHz, acetone-*d6*) δ 6.39 (dd, *J* = 6.1, 2.4 Hz, 1H), 6.28 (s, 1H), 6.12 (dt, *J* = 2.3, 0.6 Hz, 1H), 5.36 (q, *J* = 2.4 Hz, 1H), 4.96 (dd, *J* = 6.1, 2.6 Hz, 1H); ^{13}C NMR (125.76 MHz, acetone-*d6*) δ 170.51, 167.89, 141.76, 114.45, 105.94, 98.51, 63.37; ESIMS *m*/*z*: [M + Na]$^+$ 176.95.

Methyl ester of xylaric acid (**9**) [10]: White amorphous solid; ^{1}H NMR (500 MHz, CD_3OD) δ 8.17 – 8.10 (m, 1H), 8.09 (dd, *J* = 5.7, 1.0 Hz, 1H), 6.40 (d, *J* = 5.7 Hz, 1H), 3.69 (s, 3H), 3.39 (s, 1H).; ^{13}C NMR (125.76 MHz, CD_3OD) data: δ 180.20, 172.52, 158.72, 156.98, 125.39, 117.06, 52.62, 31.43; ESIMS *m*/*z*: [M+Na] 191.14.

Figure S1. GNPS Feature-based Molecular Networks of *T. cacao* associated endophytes *C. rosea*.

Pink shadow denotes dereplicated cluster compounds and adducts observed. Node color shows as m/z gradient of each feature detected.

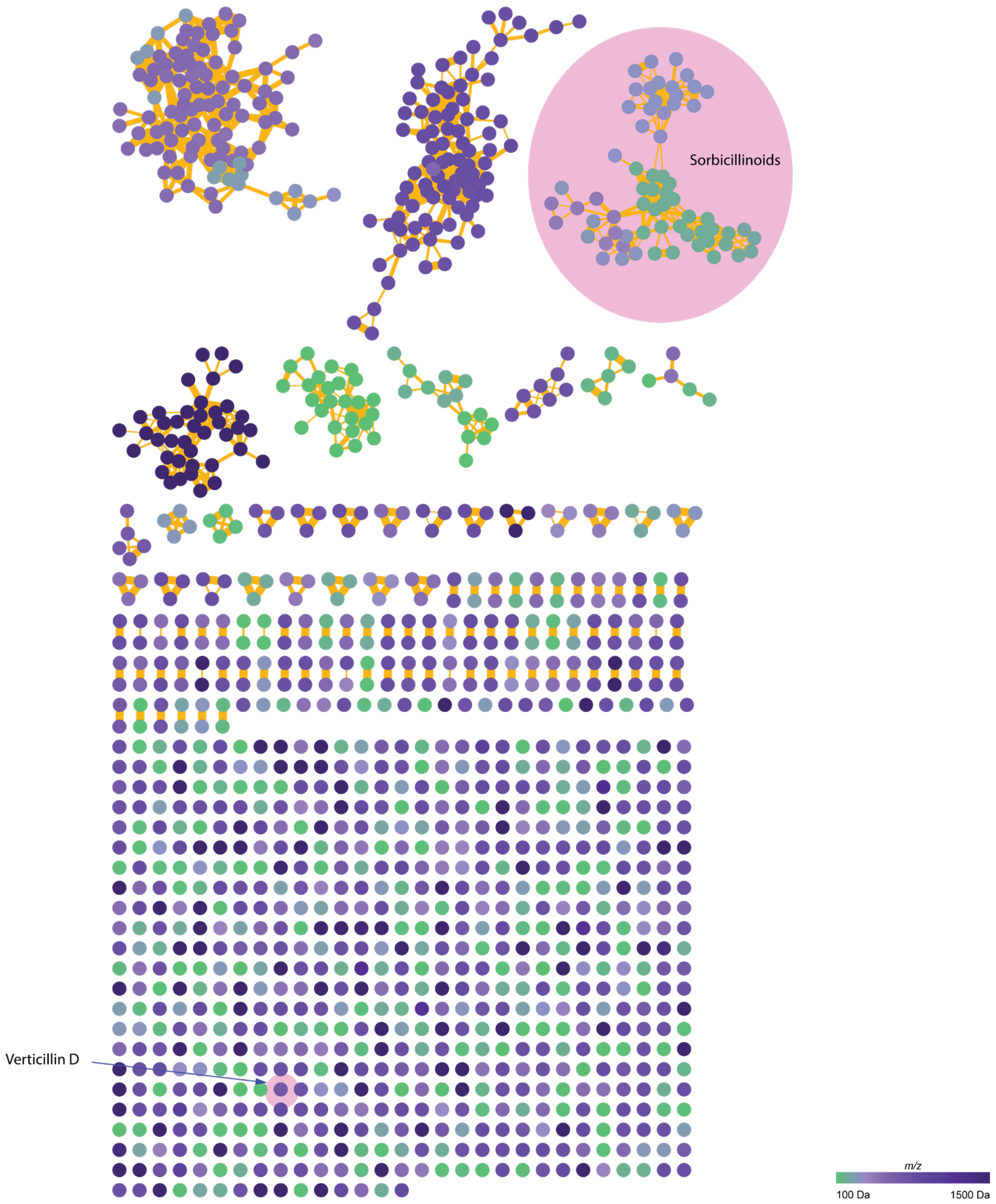

Figure S2. GNPS Feature-based Molecular Networks of *T. cacao* associated endophytes *W. zeylanica*.

Pink shadow denotes dereplicated cluster compounds and adducts observed. Node color shows as m/z gradient of each feature detected.

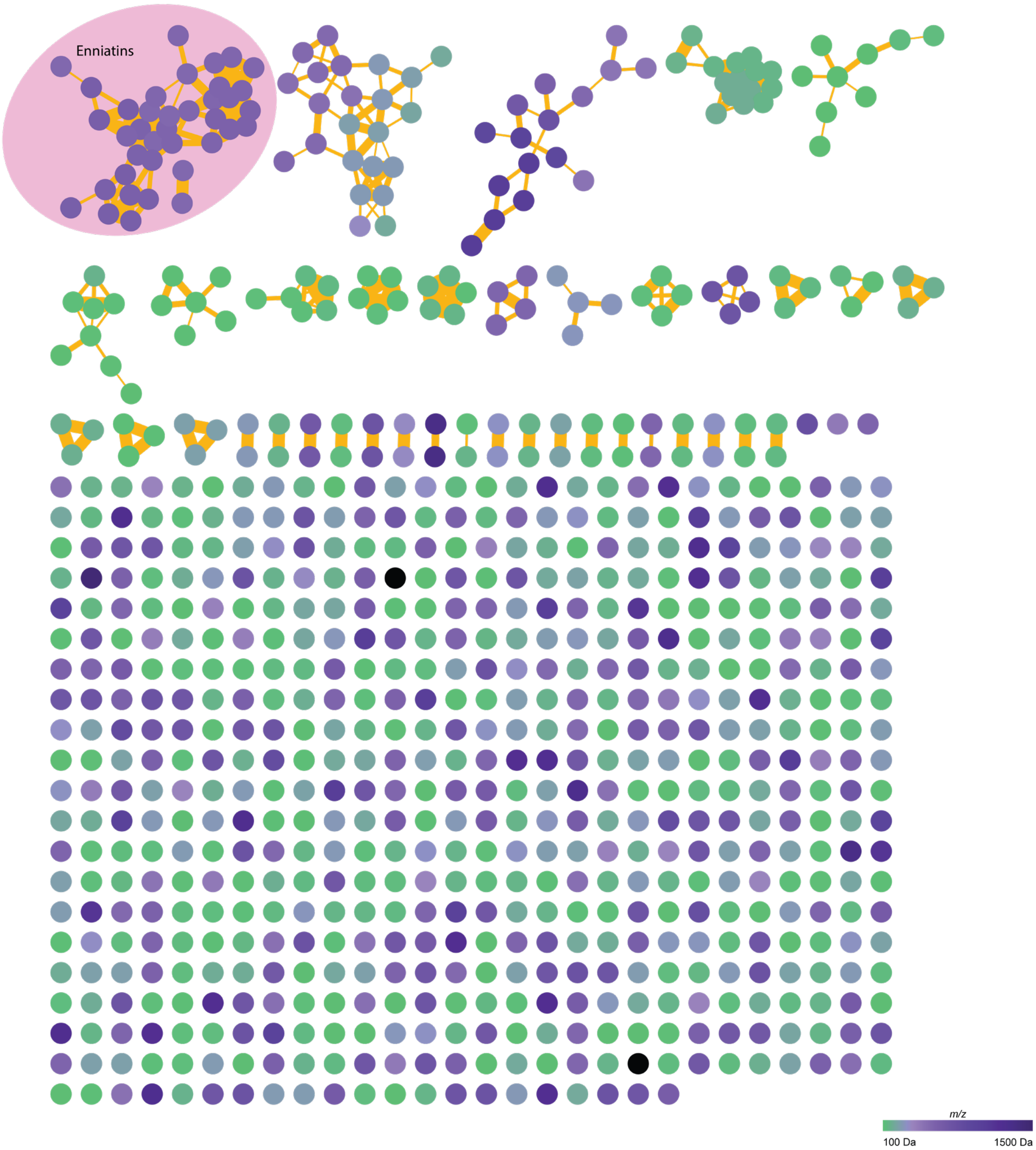

Figure S3. GNPS Feature-based Molecular Networks of *C. arabica* associated endophytes *Multiguttulispora sp.*

Pink shadow denotes dereplicated cluster compounds and adducts observed. Node color shows as m/z gradient of each feature detected.

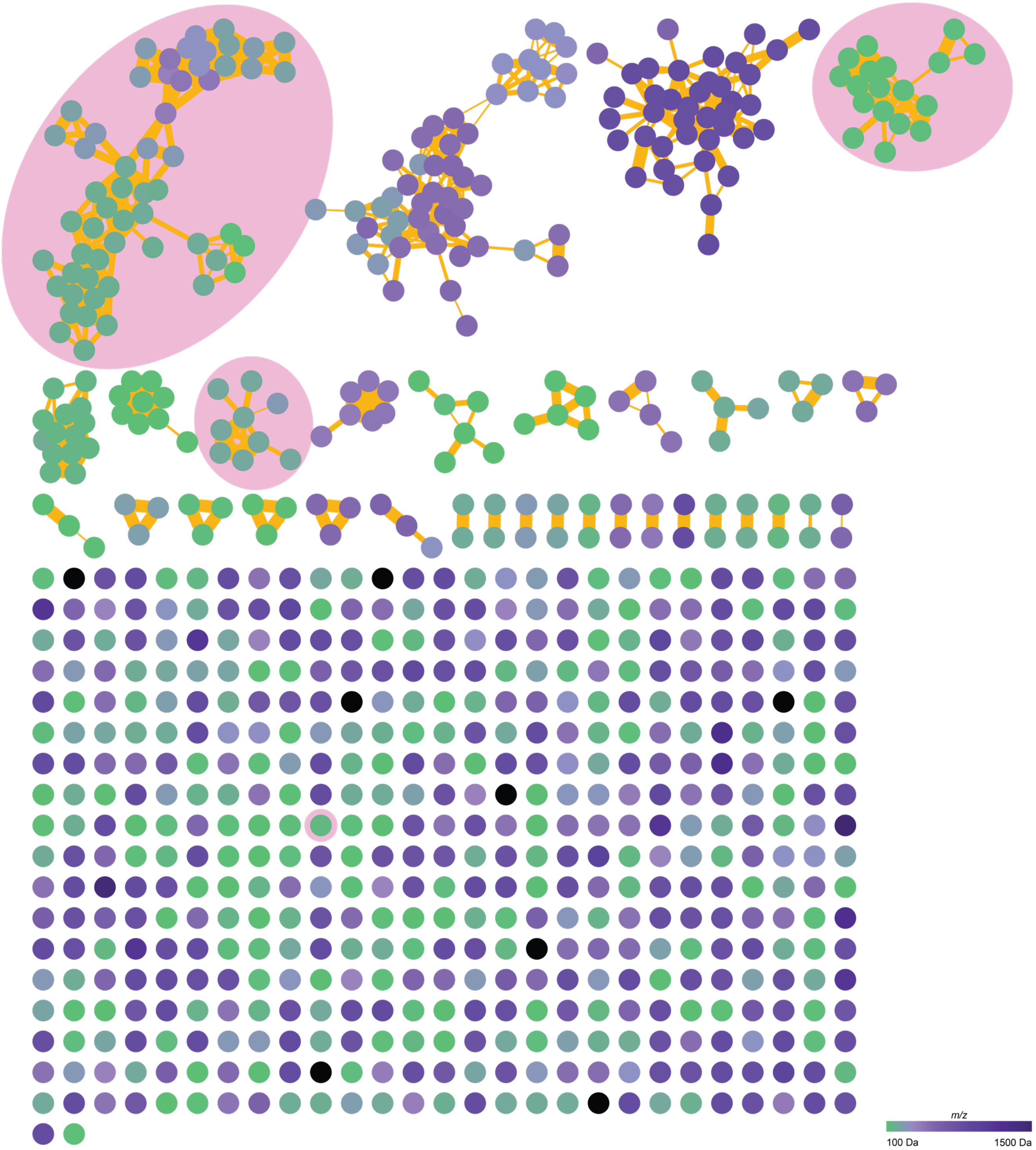

Figure S4. GNPS Feature-based Molecular Networks of *C. arabica* associated endophytes *X. grammica*.

Pink shadow denotes dereplicated cluster compounds and adducts observed. Node color shows as m/z gradient of each feature detected.

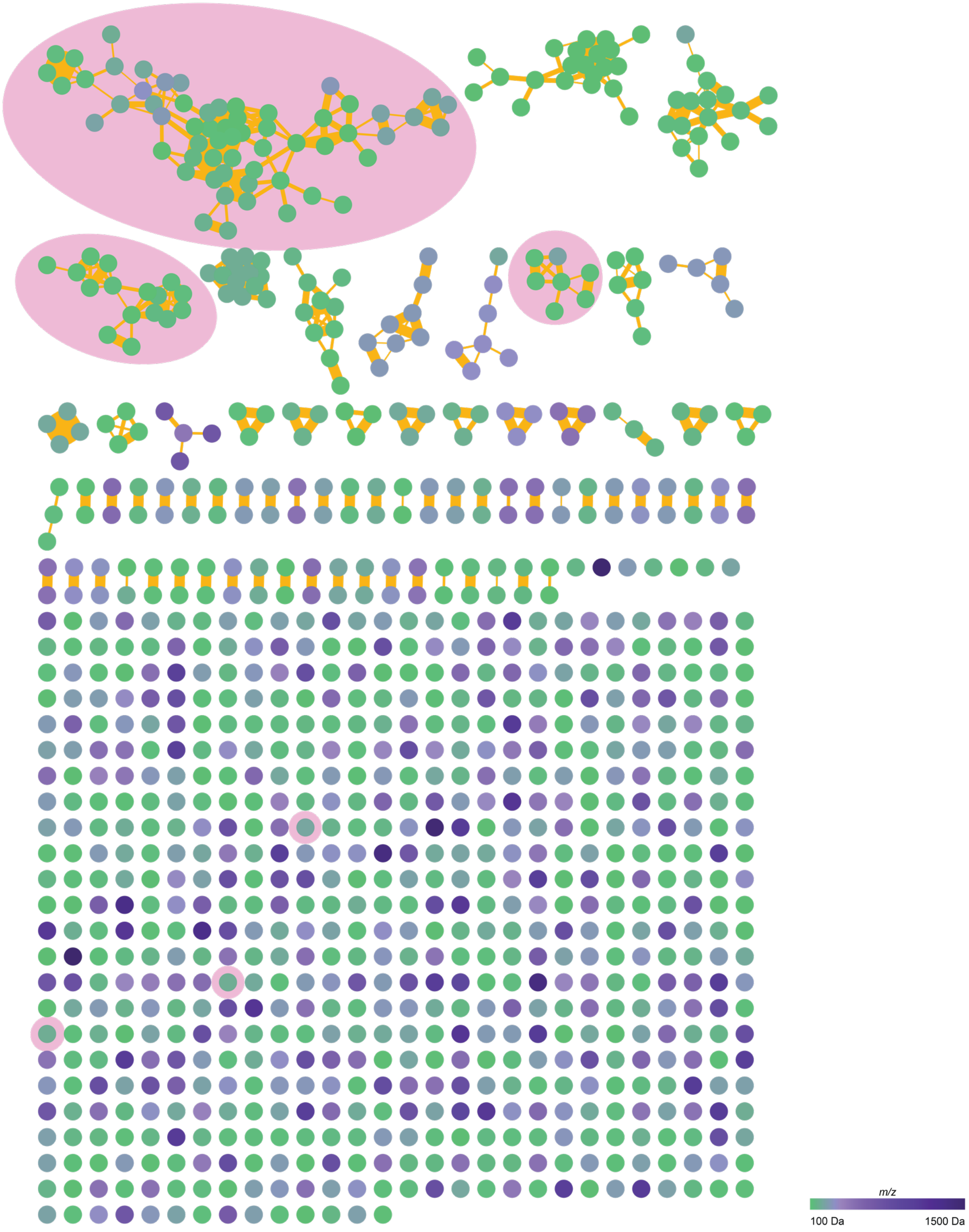

Figure S5. Metabolomics spectrum resolver mirror plot of verticillin D (**1**) and GNPS Library Spectrum CCMSLIB00000478454 at the Universal Spectrum Identifier (USI).

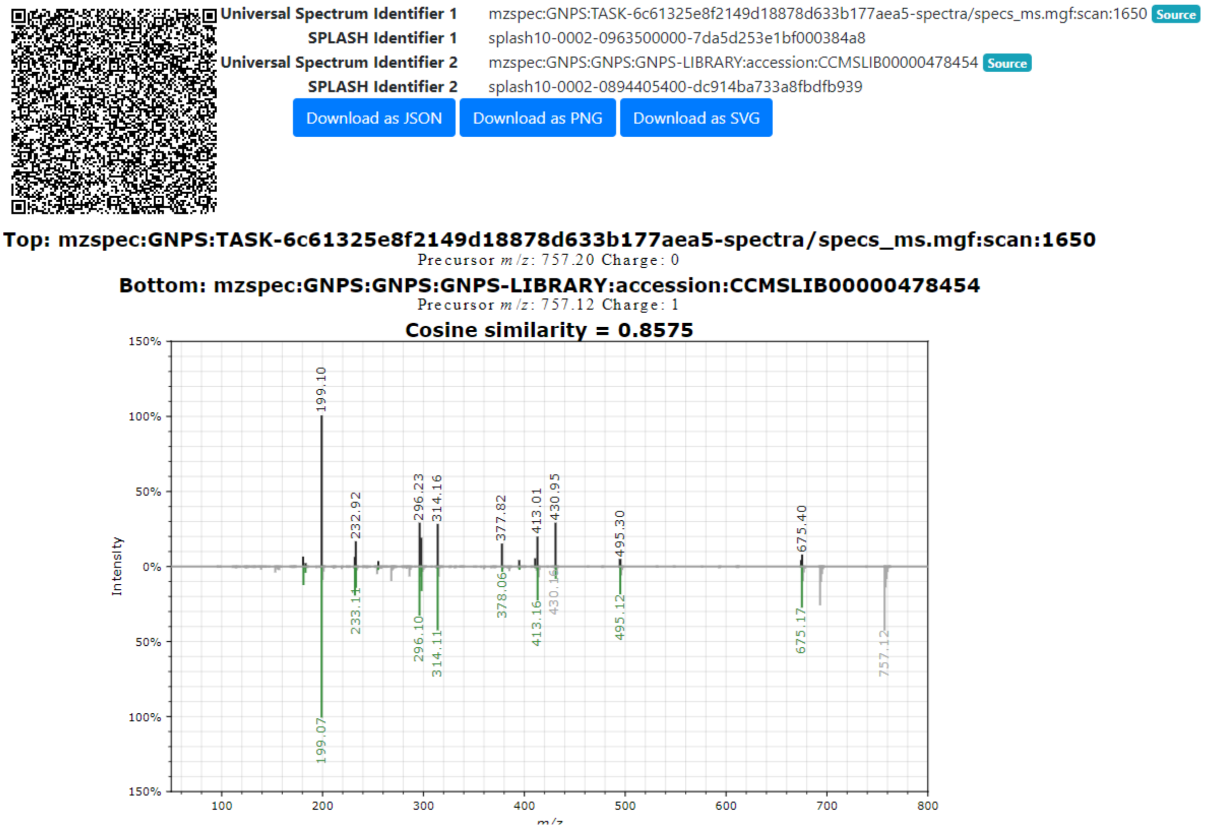

Figure S6. Metabolomics spectrum resolver mirror plot of bislongiquinolide (**10**) and GNPS Library Spectrum CCMSLIB00004712865 at the Universal Spectrum Identifier (USI).

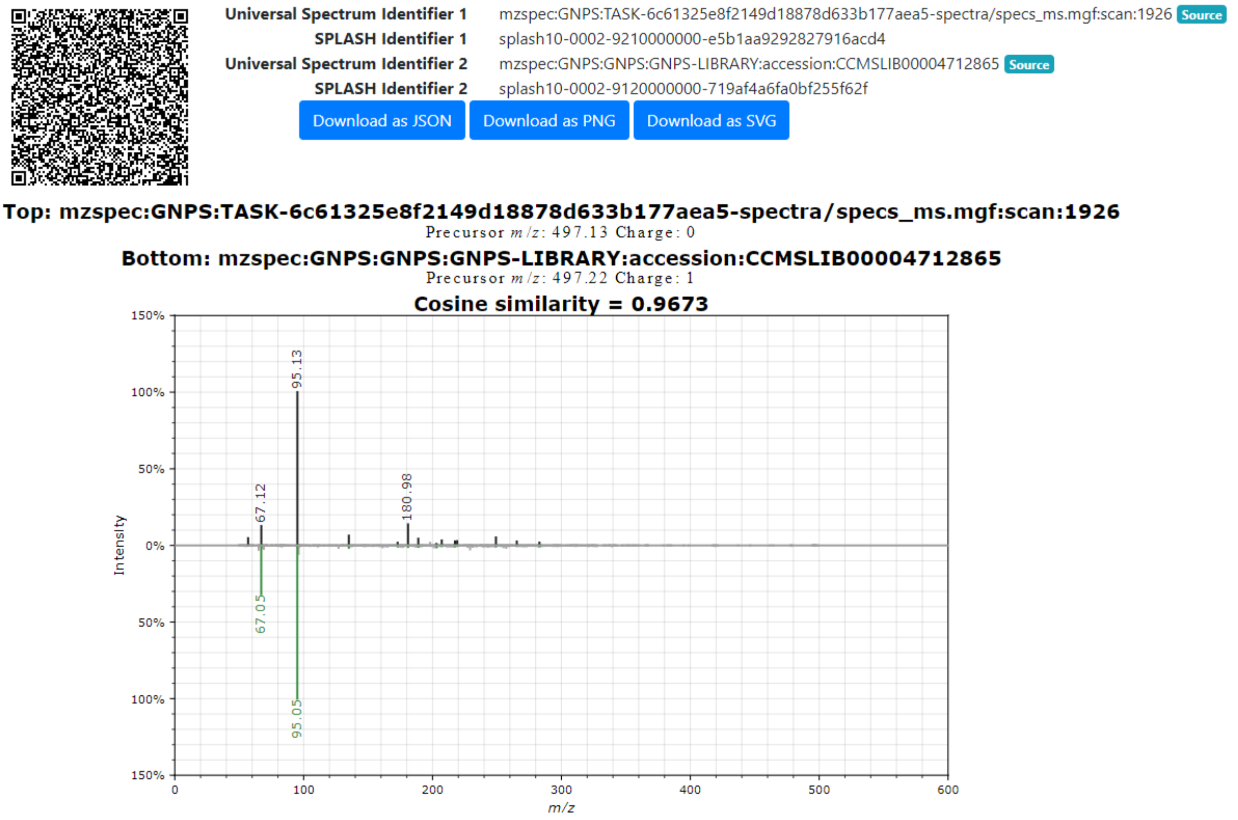

Figure S7. Metabolomics spectrum resolver mirror plot of trichodimerol (**11**) and GNPS Library Spectrum CCMSLIB00000855732 at the Universal Spectrum Identifier (USI).

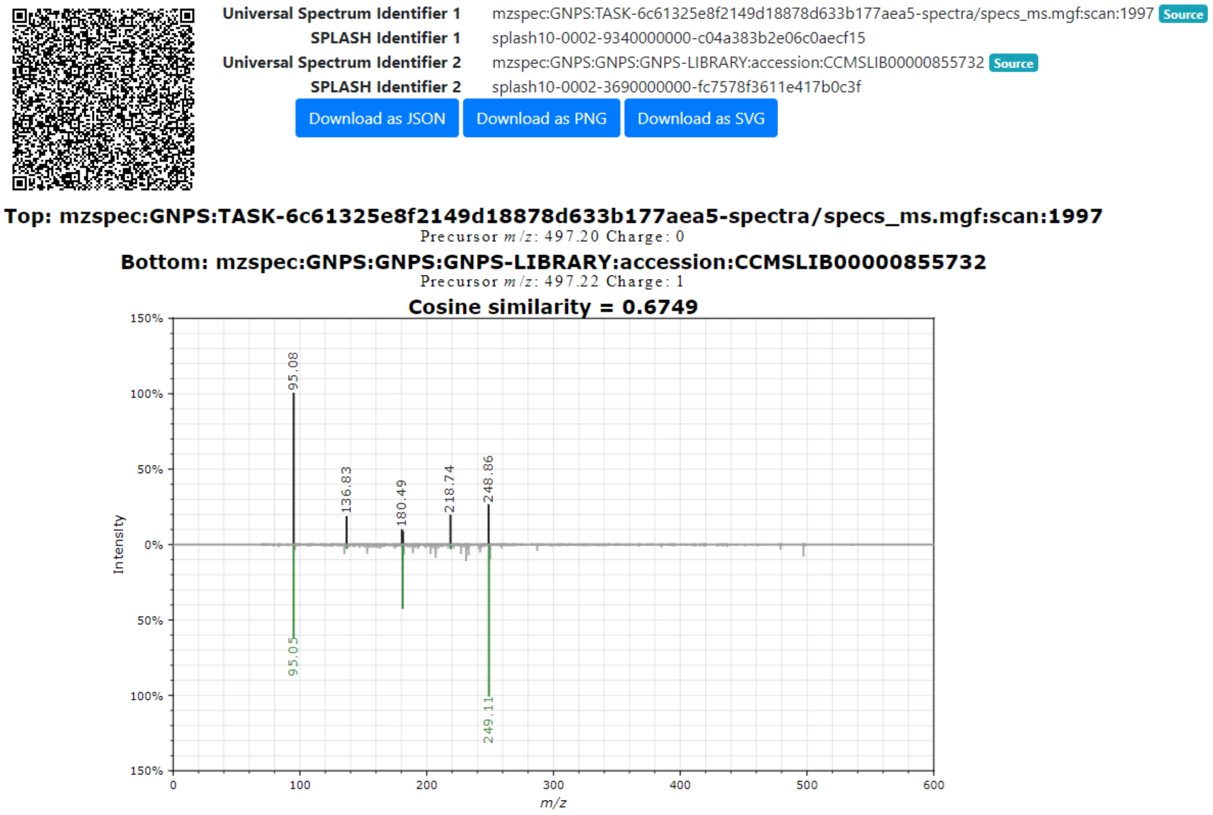

Figure S8. Metabolomics spectrum resolver mirror plot of dihydrotrichodimerol (**12**) and GNPS Library Spectrum CCMSLIB00000851924 at the Universal Spectrum Identifier (USI).

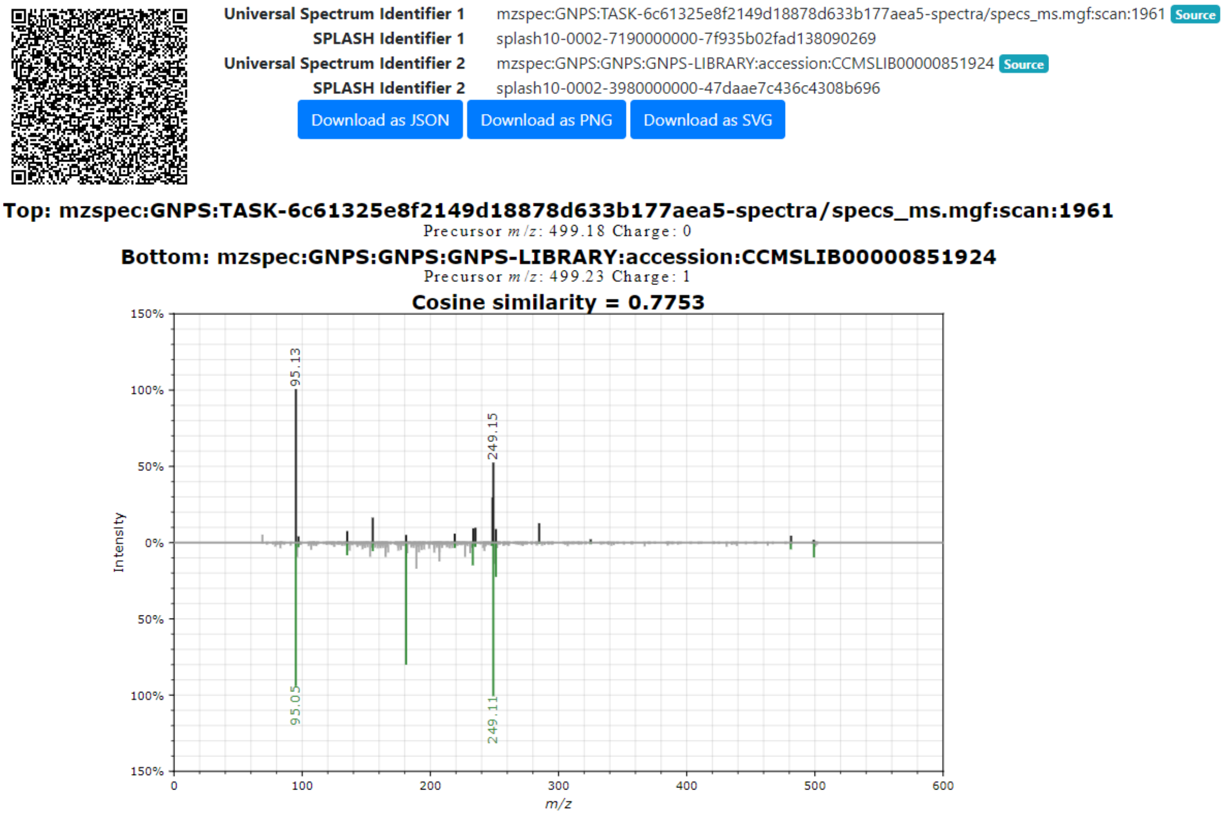

Figure S9. Metabolomics spectrum resolver mirror plot of enniatin B (**2**) and GNPS Library Spectrum CCMSLIB00005727731 at the Universal Spectrum Identifier (USI).

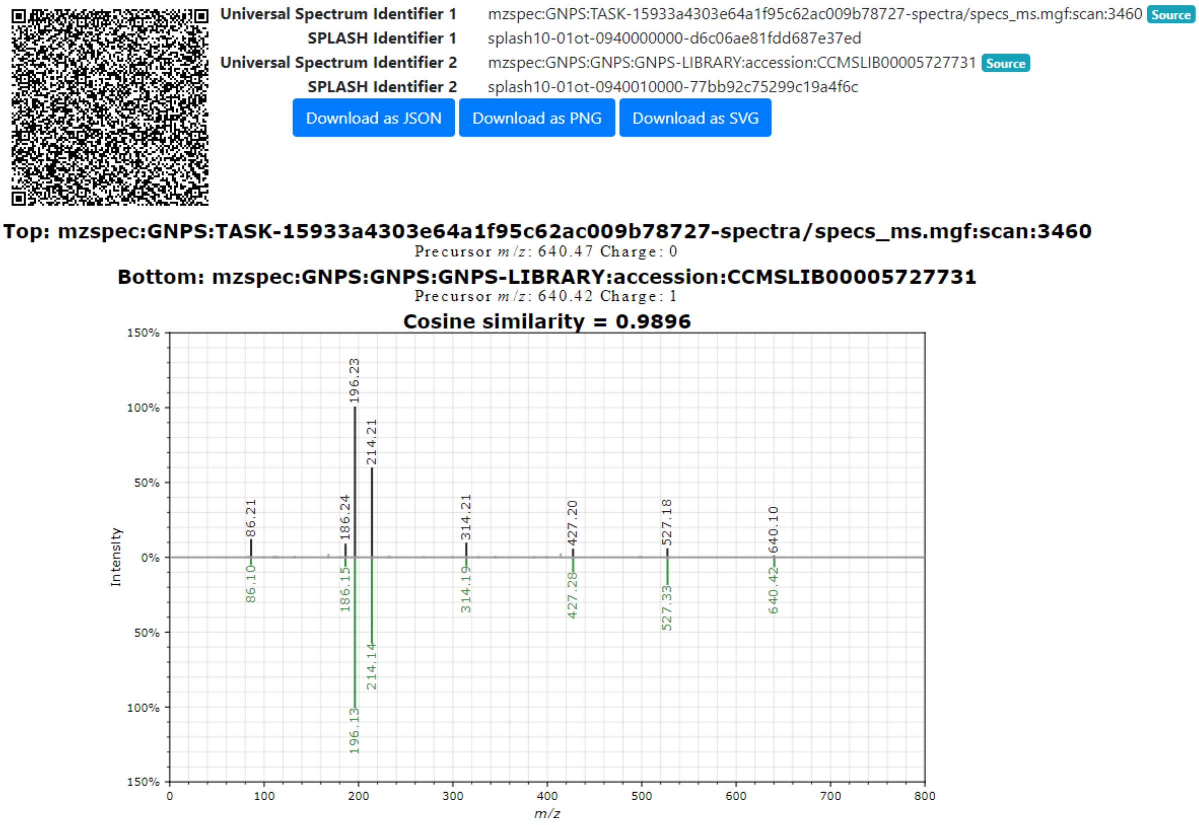

Figure S10. Metabolomics spectrum resolver mirror plot of enniatin B4 (**3**) and GNPS Library Spectrum CCMSLIB00000577644 at the Universal Spectrum Identifier (USI).

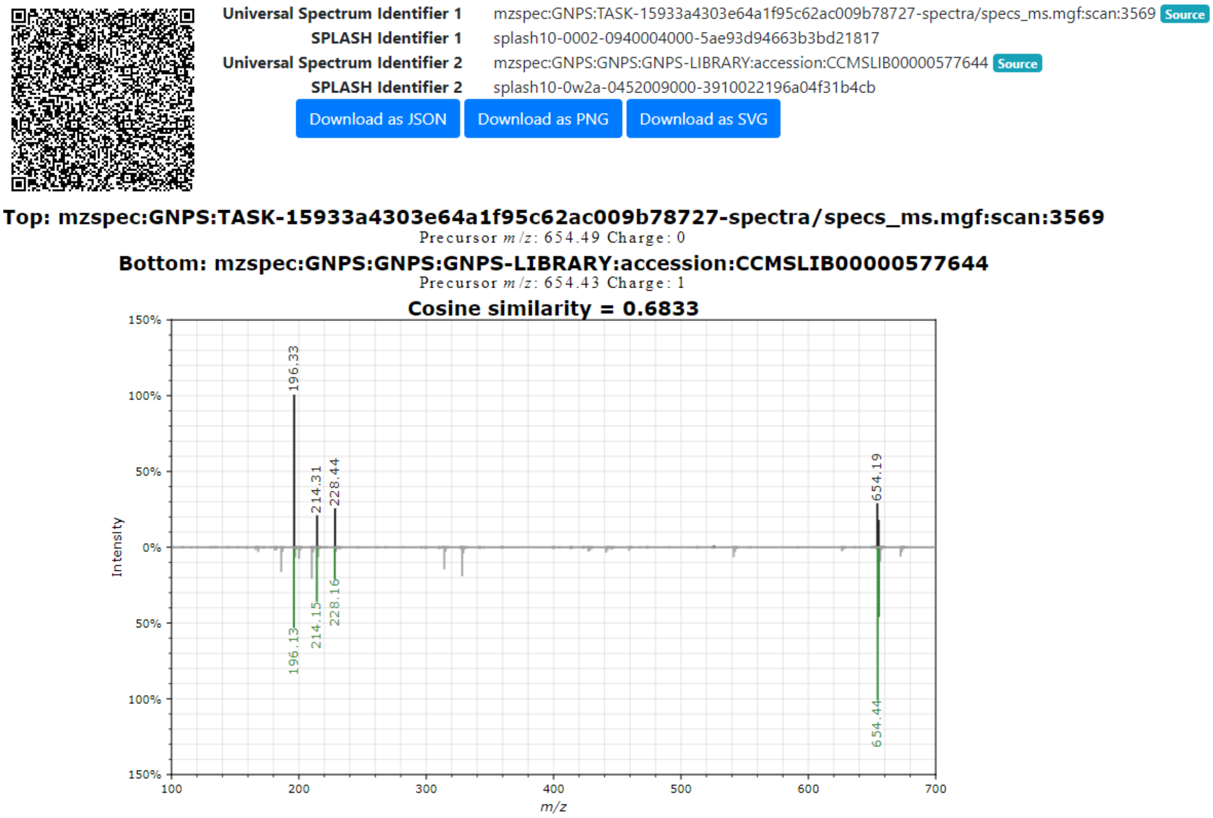

Figure S11. Metabolomics spectrum resolver mirror plot of enniatin A1 (**4**) and GNPS Library Spectrum CCMSLIB00005727903 at the Universal Spectrum Identifier (USI).

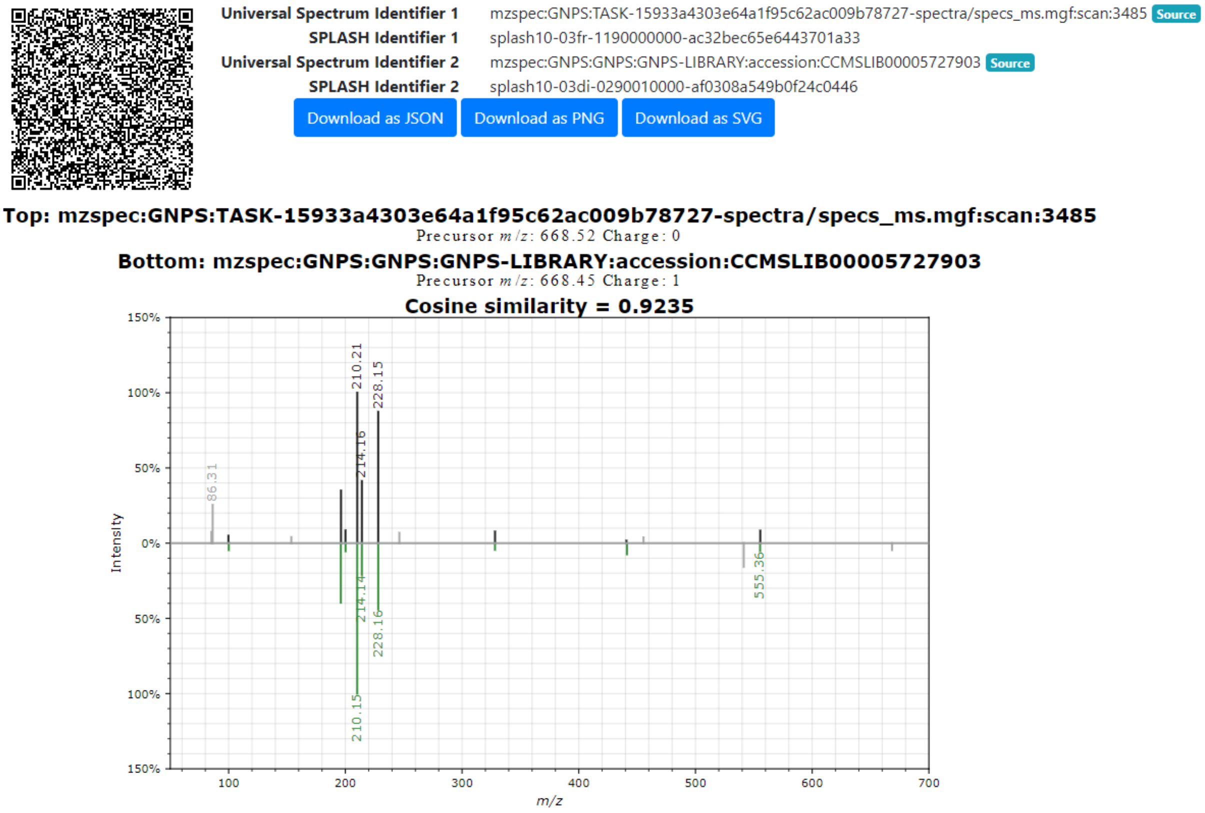

Figure S12. Metabolomics spectrum resolver mirror plot of enniatin J1 (**13**) and GNPS Library Spectrum CCMSLIB00000577703 at the Universal Spectrum Identifier (USI).

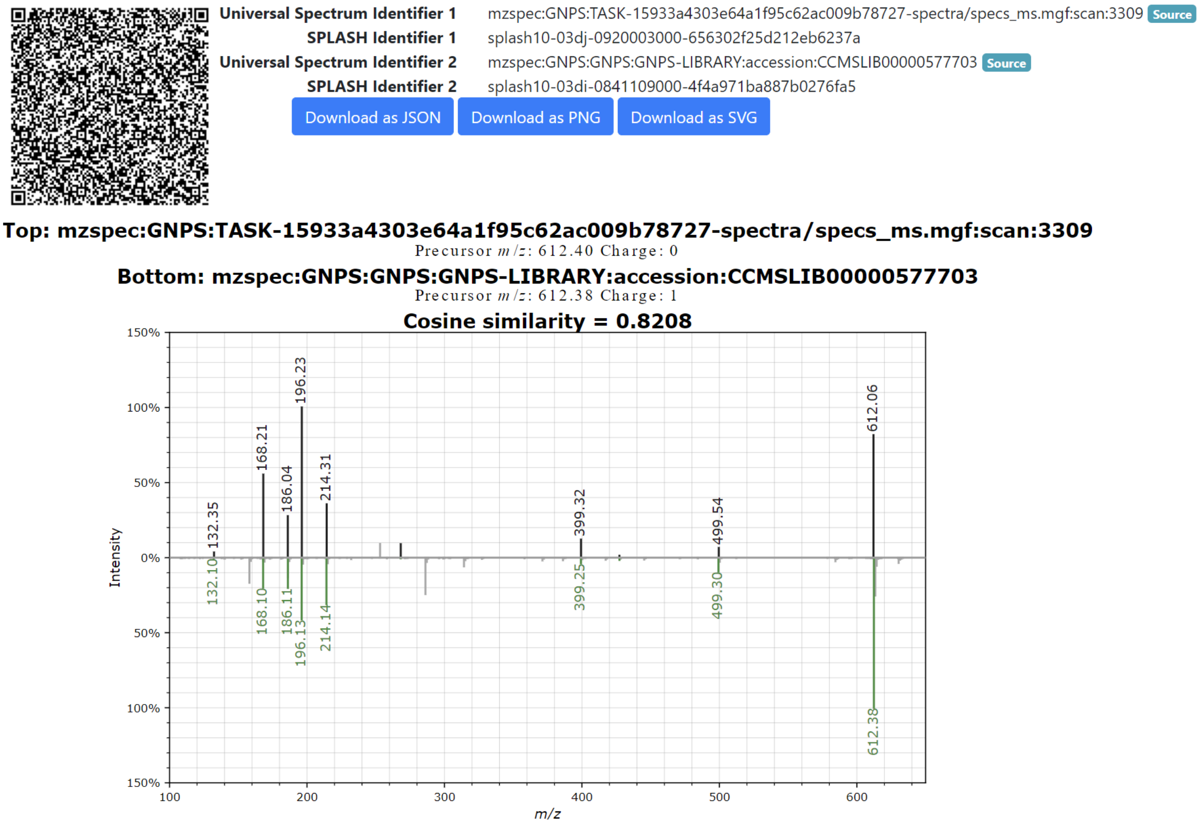

Figure S13. Metabolomics spectrum resolver mirror plot of enniatin J2 (**14**) and GNPS Library Spectrum CCMSLIB00000577620 at the Universal Spectrum Identifier (USI).

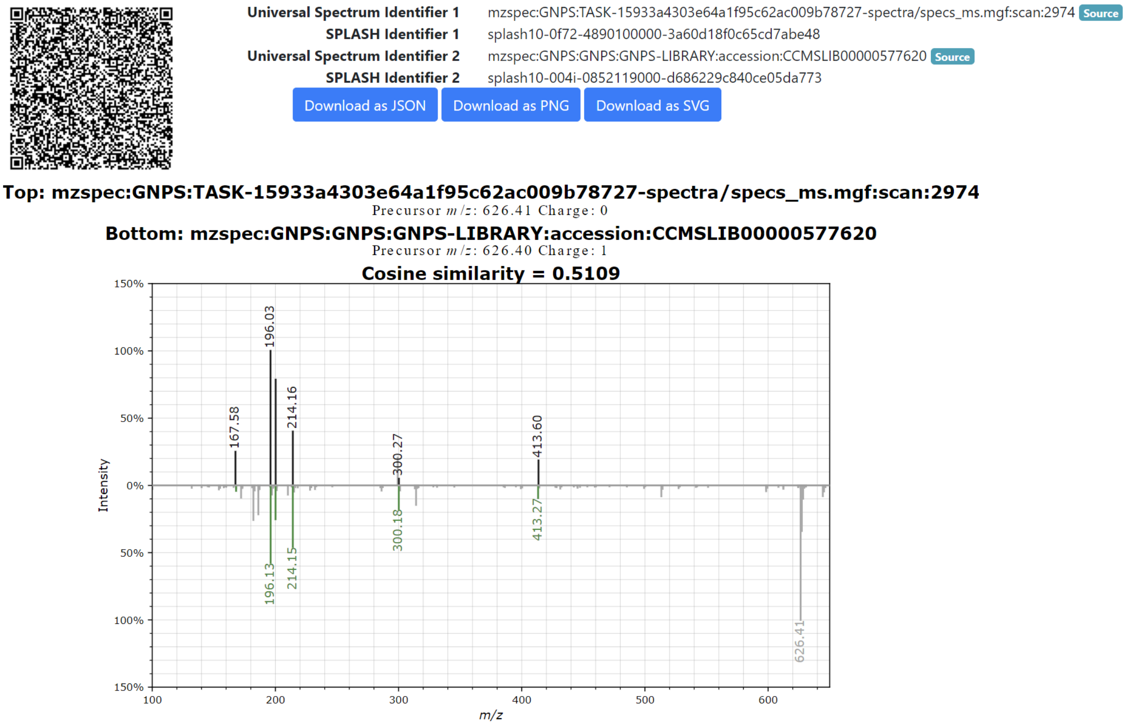

Figure S14. Metabolomics spectrum resolver mirror plot of enniatin B2 (**15**) and GNPS Library Spectrum CCMSLIB00000577662 at the Universal Spectrum Identifier (USI).

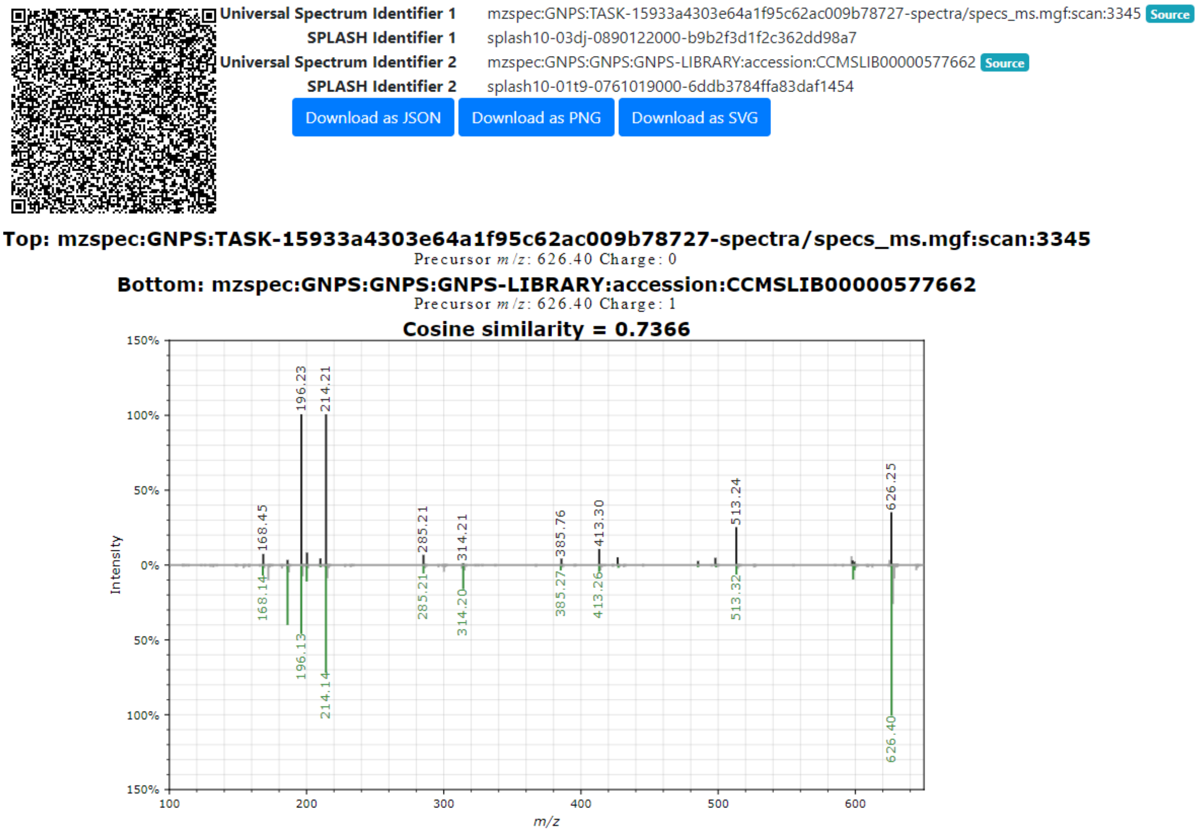

Figure S15. Metabolomics spectrum resolver mirror plot of enniatin B3 (**16**) and GNPS Library Spectrum CCMSLIB00000577824 at the Universal Spectrum Identifier (USI).

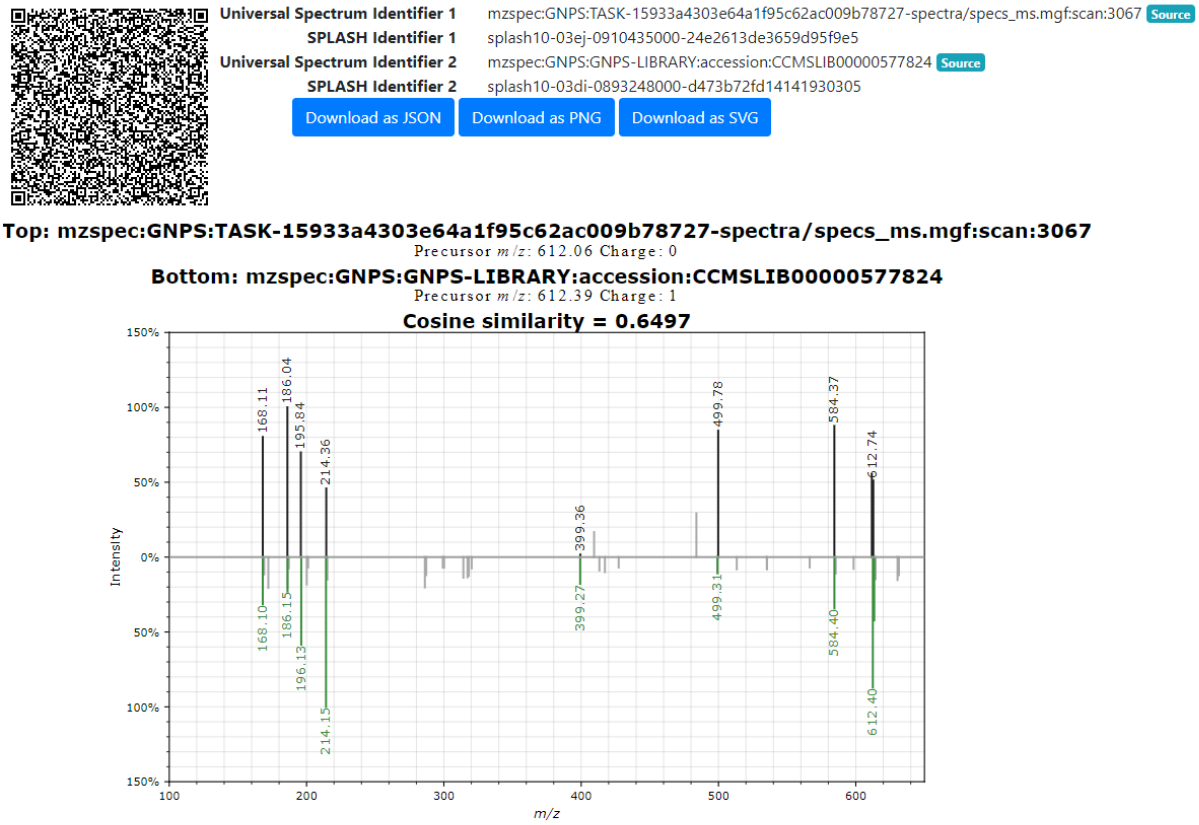

Figure S16. Metabolomics spectrum resolver mirror plot of trans-3,4-hydroxymellein (**5**) and GNPS Library Spectrum CCMSLIB00000478502 (4-hydroxymellein) at the Universal Spectrum Identifier (USI).

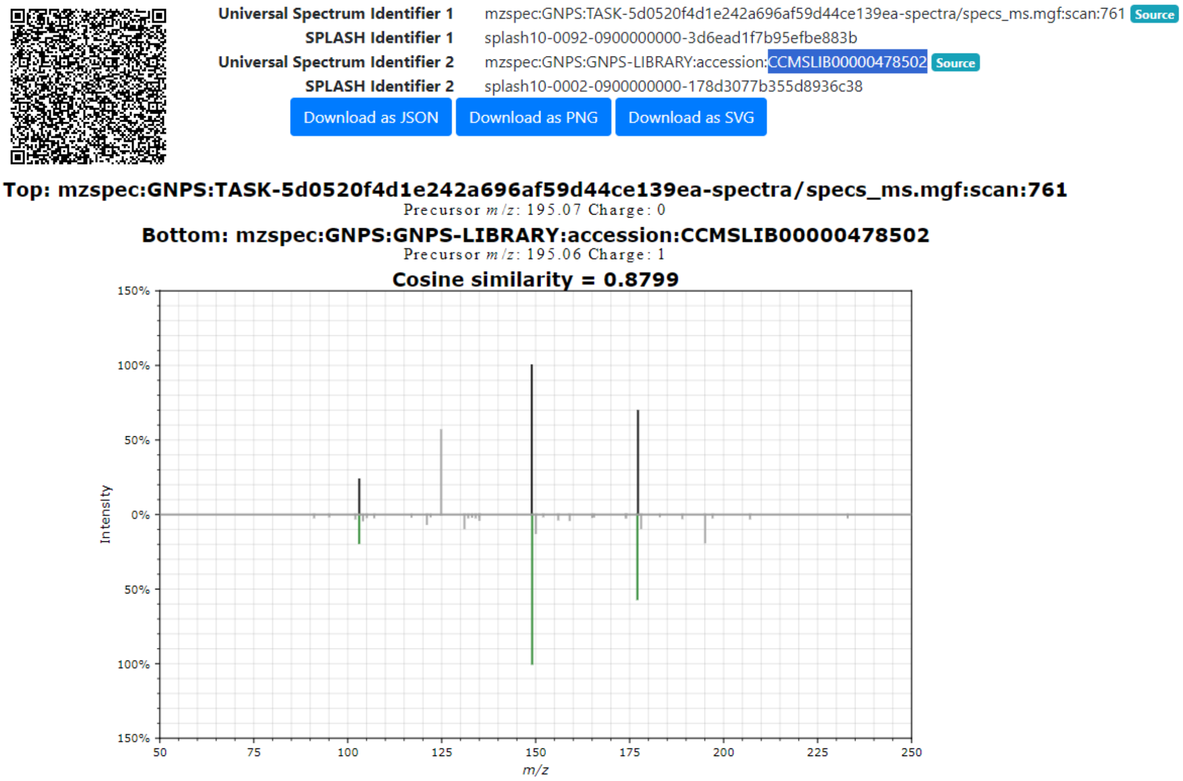

Figure S17. Metabolomics spectrum resolver mirror plot of mellein (**17**) and GNPS Library Spectrum CCMSLIB00005727561 at the Universal Spectrum Identifier (USI).

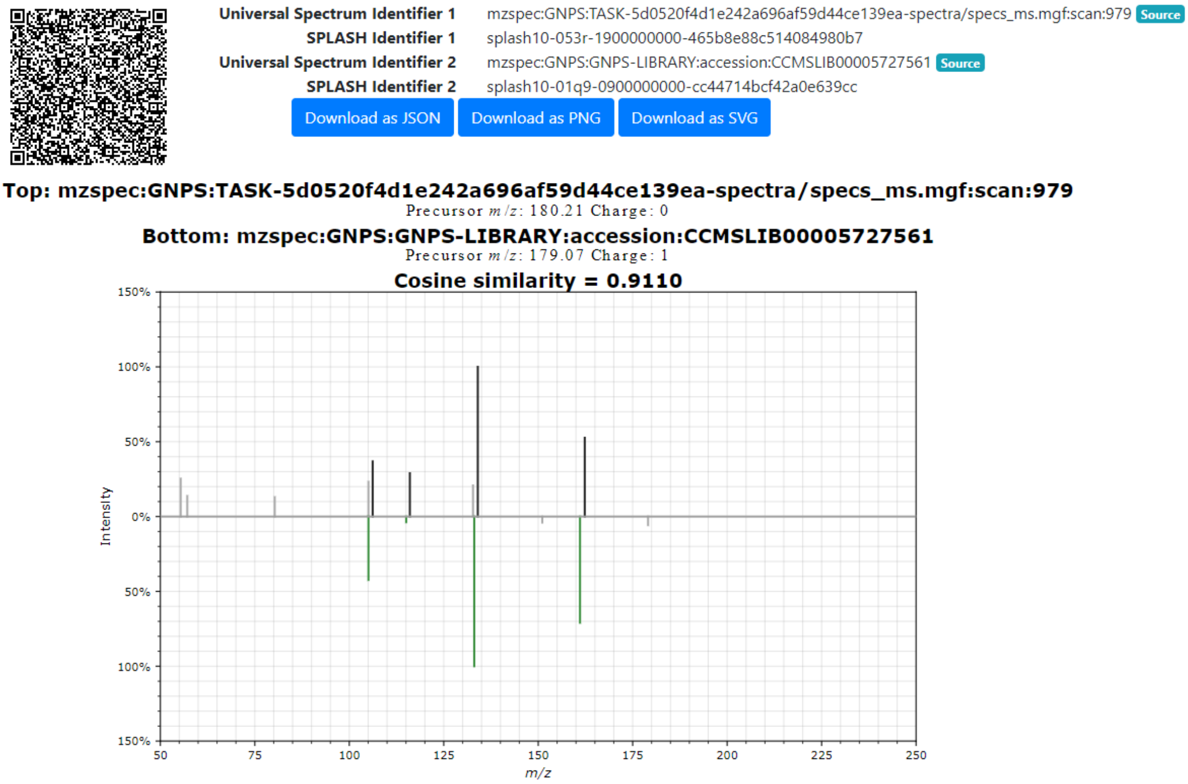

Figure S18. Metabolomics spectrum resolver mirror plot of fusaric acid (**18**) and GNPS Library Spectrum CCMSLIB00005743636 at the Universal Spectrum Identifier (USI).

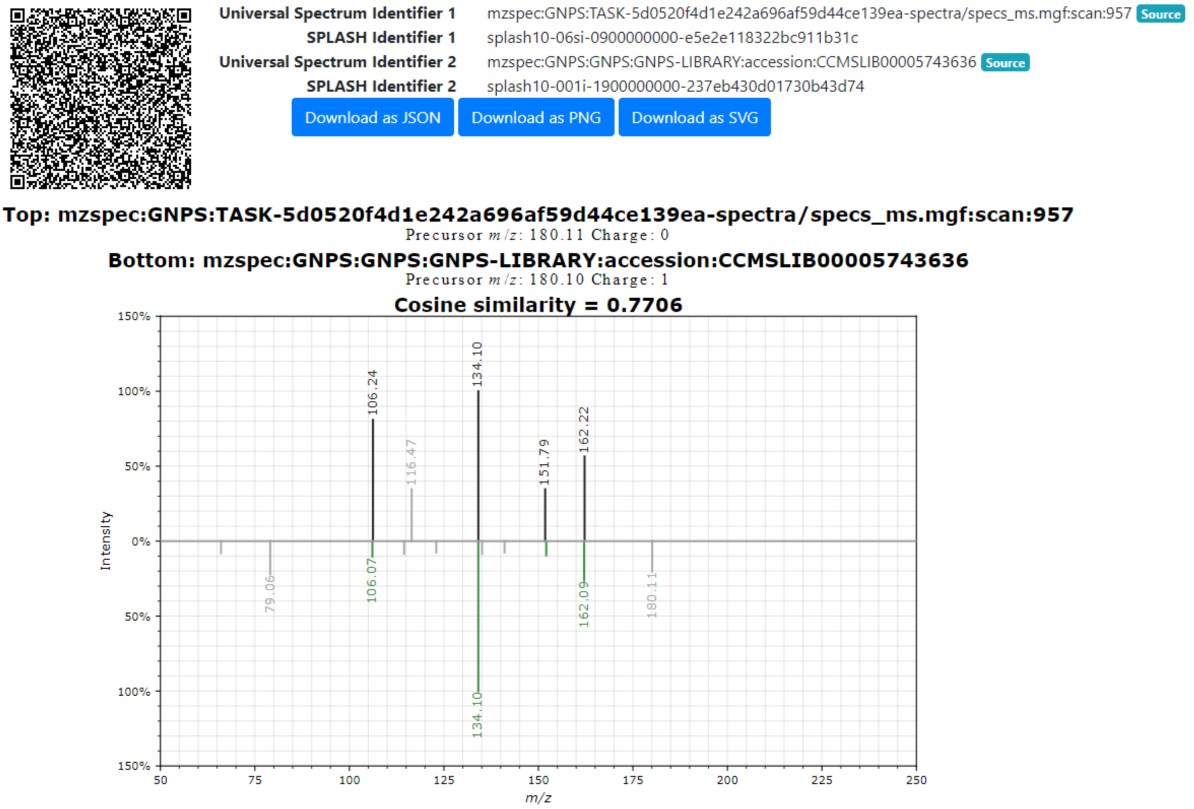

Figure S19. Metabolomics spectrum resolver mirror plot of flavipucine (**6**) and GNPS Library Spectrum CCMSLIB00012725756 at the Universal Spectrum Identifier (USI).

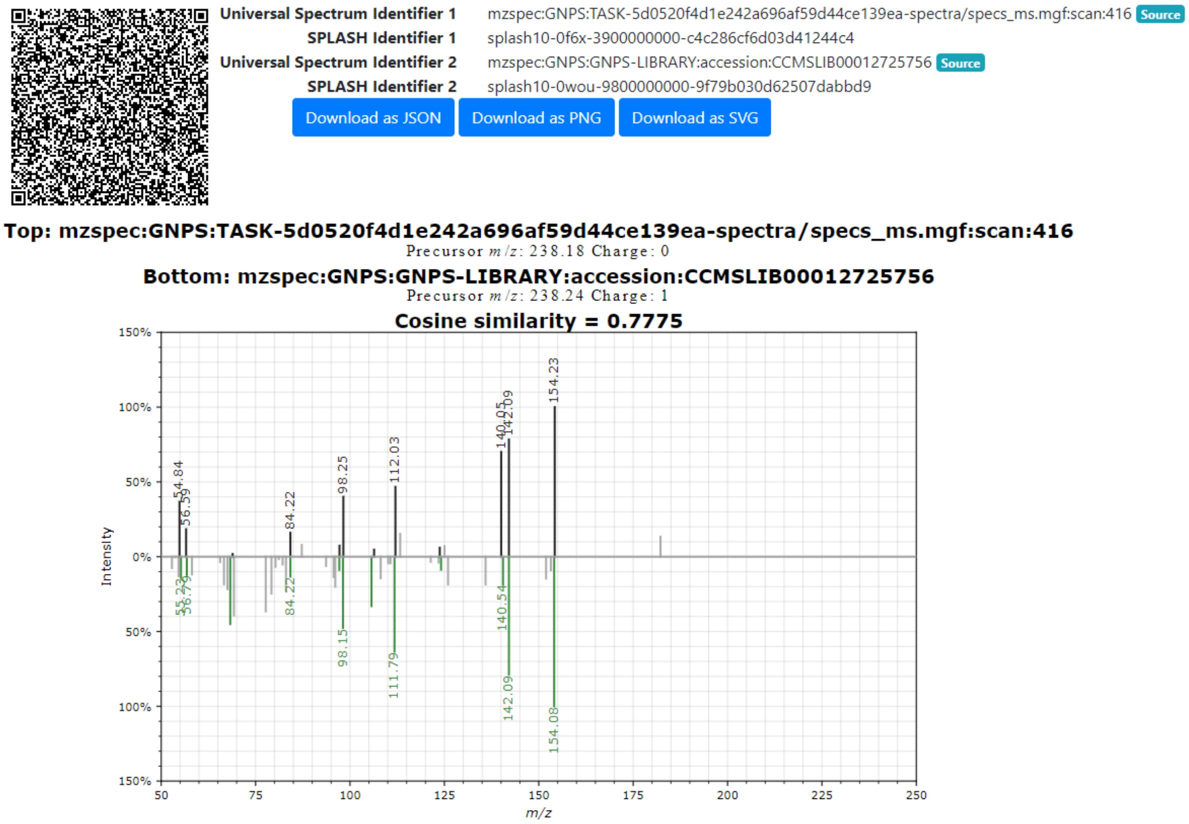

Figure S20. Metabolomics spectrum resolver mirror plot of isoflavipucine (**19**) and peak table reported by Findlay et al 1977 as mgf format, uploaded to GNPS and imported to the Universal Spectrum Identifier (USI)

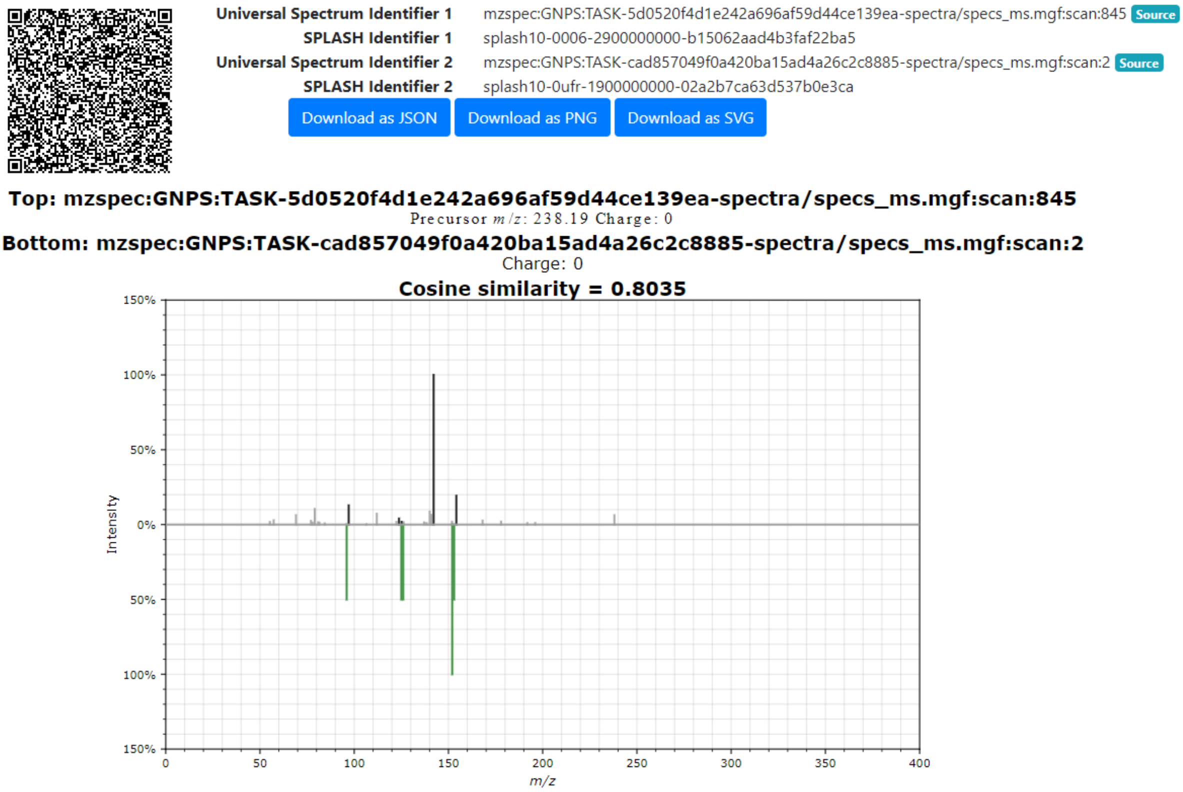

Findlay, J. A., Krepinsky, J., Shum, A., Casinovi, C. G. & Radics, L. The structure of isoflavipucine. *Can J Chem.* **55**, 600–603 (1977).

Figure S21. Metabolomics spectrum resolver mirror plot of dihydroisoflavipucine (**20**) and GNPS Library Spectrum CCMSLIB00000854979 at the Universal Spectrum Identifier (USI).

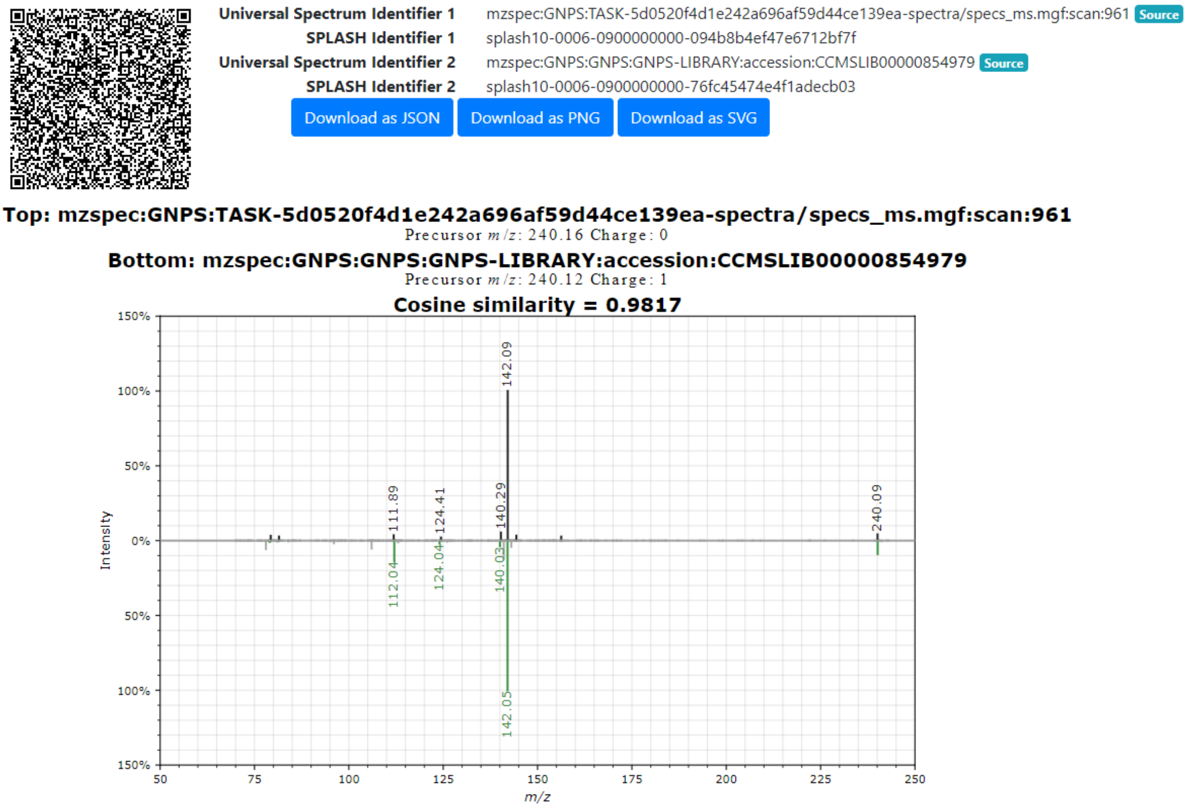

Figure S22. Metabolomics spectrum resolver mirror plot of the tautomeric methoxyl acetal of isoflavipucine (**21**) and GNPS Library Spectrum CCMSLIB00004710594 at the Universal Spectrum Identifier (USI).

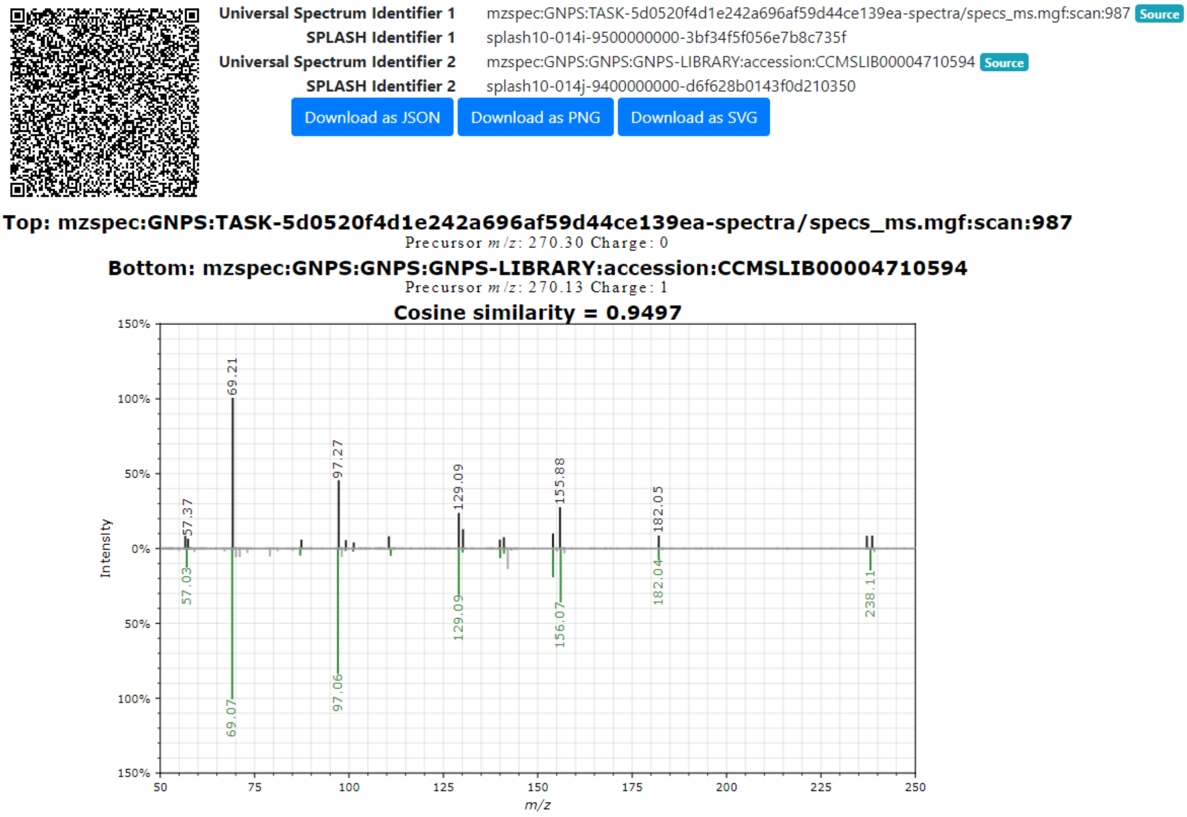

Figure S23. Metabolomics spectrum resolver mirror plot of xylaric acid (**7**) and GNPS Library Spectrum CCMSLIB00012474990 at the Universal Spectrum Identifier (USI).

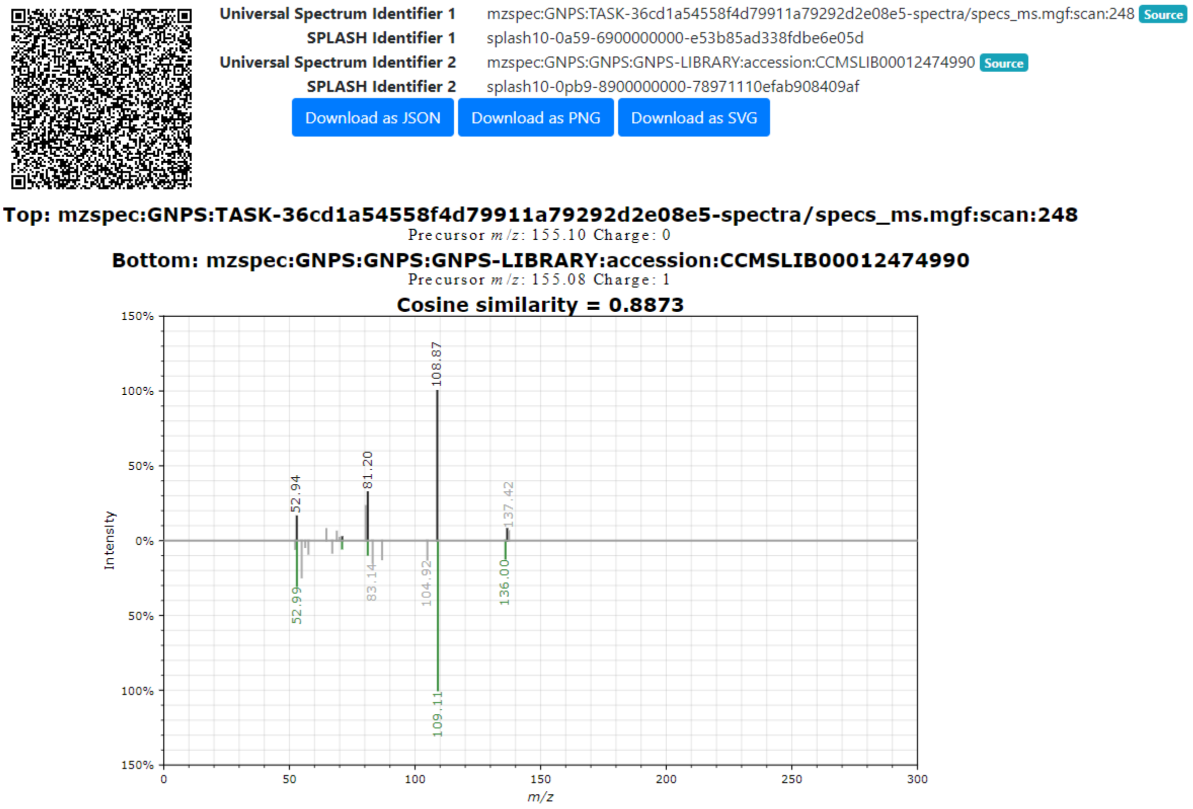

Figure S24. Metabolomics spectrum resolver mirror plot of grammicin (**8**) and GNPS Library Spectrum CCMSLIB00012474133 at the Universal Spectrum Identifier (USI).

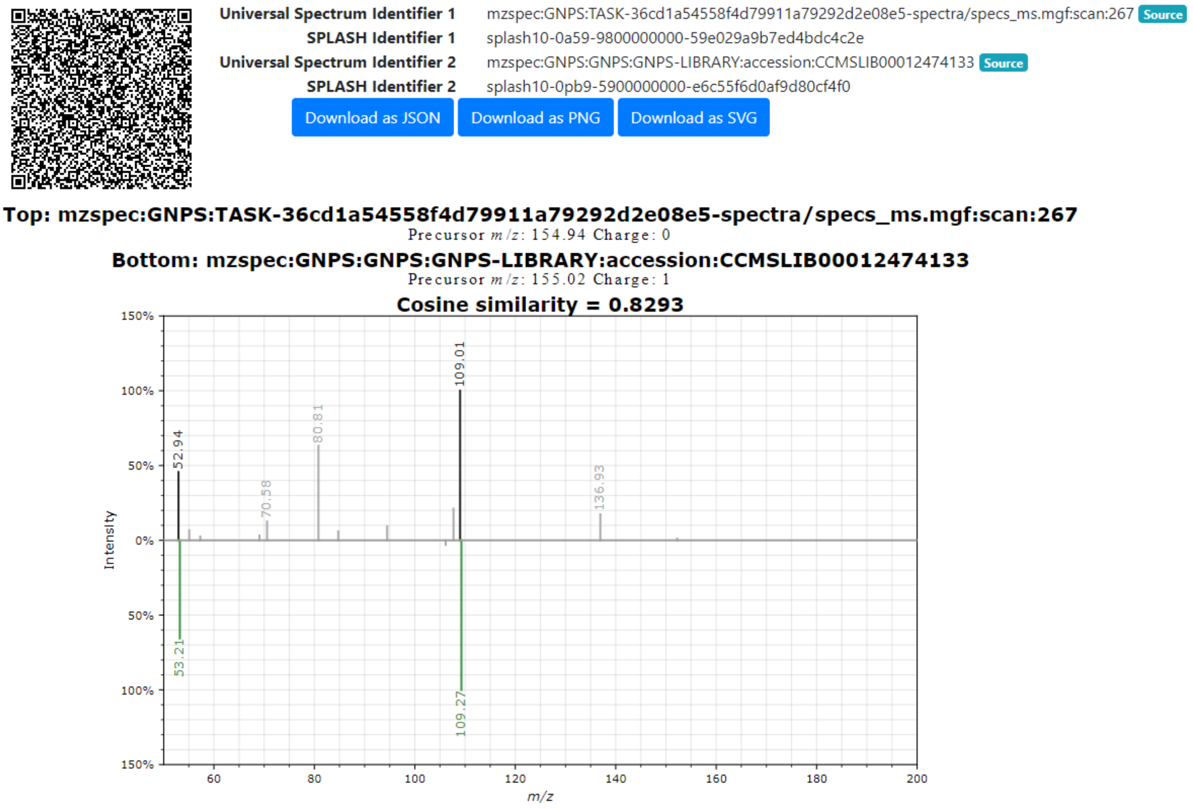

Figure S25. Metabolomics spectrum resolver mirror plot of the methyl ester of xylaric acid (**9**) and GNPS Library Spectrum CCMSLIB00012474993 at the Universal Spectrum Identifier (USI).

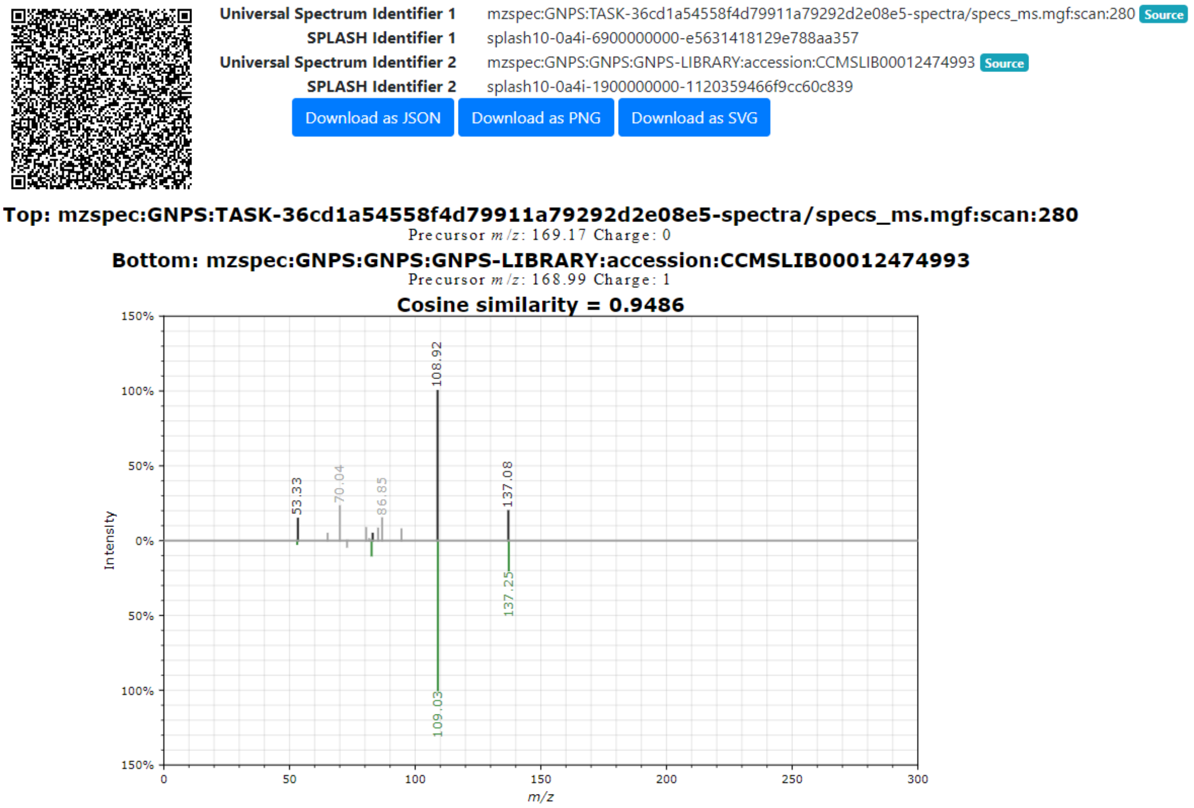

Figure S26. Metabolomics spectrum resolver mirror plot of linoleic acid (**22**) and GNPS Library Spectrum CCMSLIB00011428768 at the Universal Spectrum Identifier (USI).

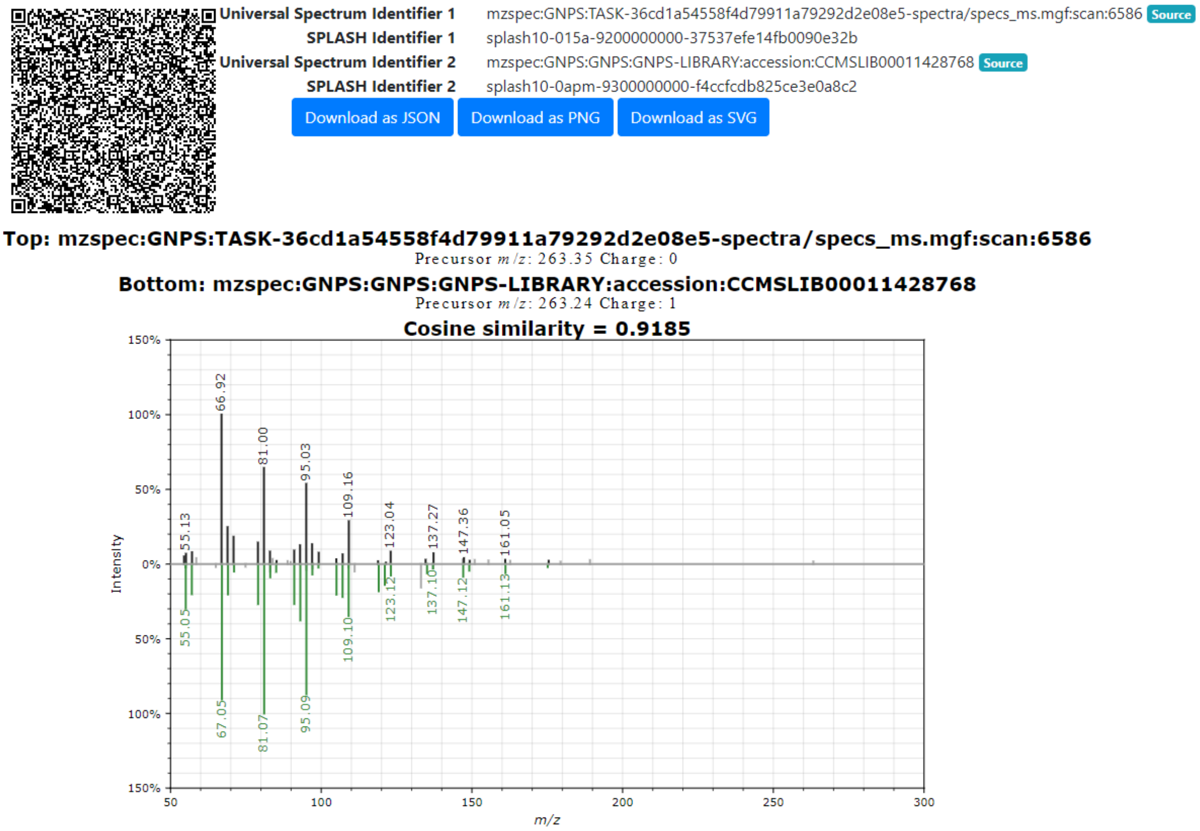

Figure S27. Metabolomics spectrum resolver mirror plot of linolenic acid (**23**) and GNPS Library Spectrum CCMSLIB00005738688 at the Universal Spectrum Identifier (USI).

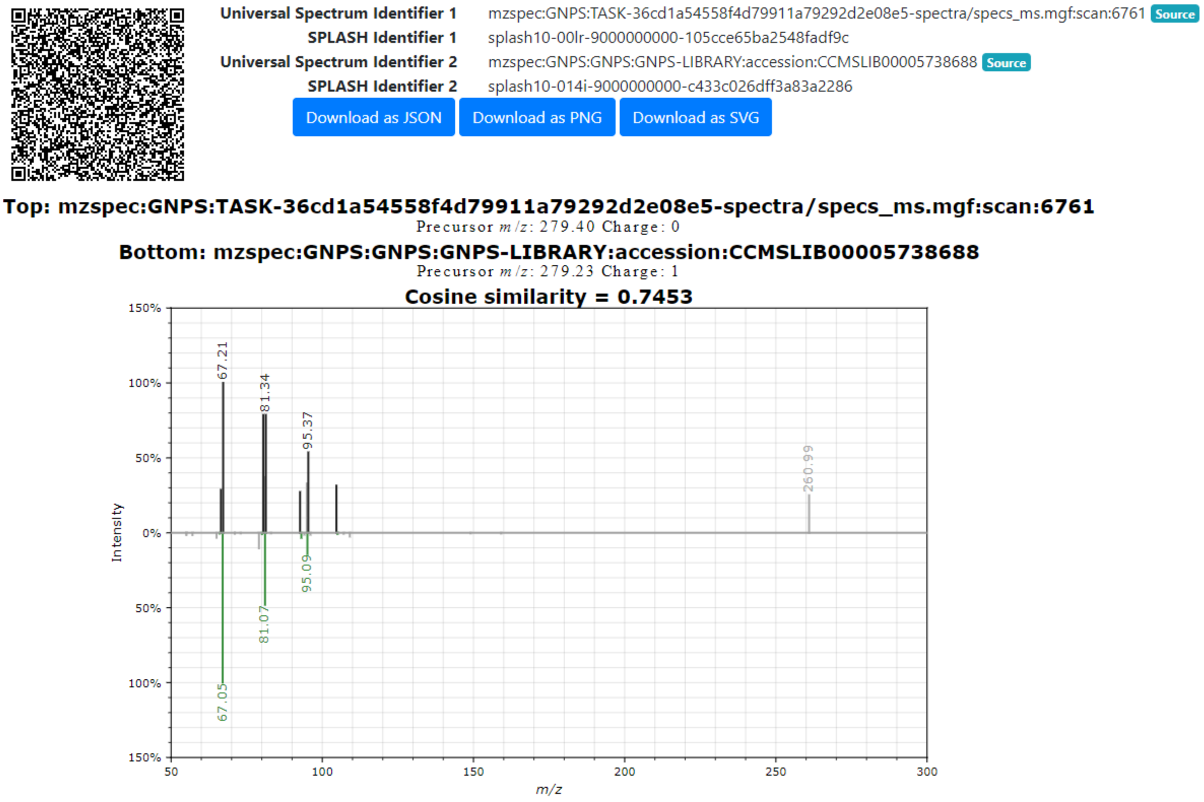

Figure S28. Homology Modeling of the enzyme *Ld*3MST with the template *Lm*3MST (1OKG).

A) Superimposition of the best *Ld*3MST model against the template *Lm*3MST with a sequence identity of 94.86% and an RMSD equal to 0.138 Å. B) Superimposition of the residues from the active site of the *Ld*3MST model against the template with an RMSD equal to 0.070 Å. Hydrogen atoms are hidden for clarity.

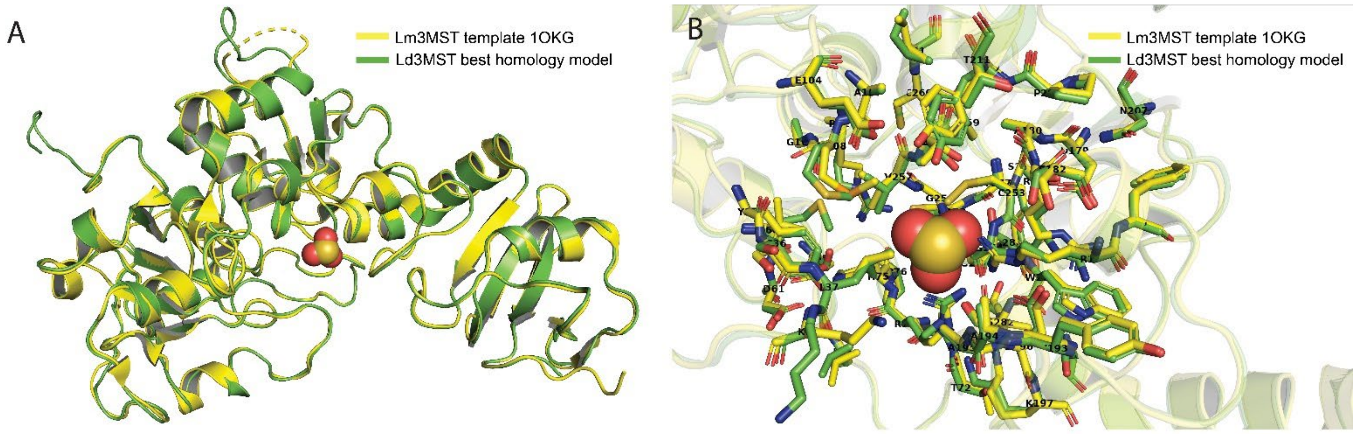

Seq. Identity = 94.86 %; RMSD = 0.138 Å (305 to 305 atoms)

RMSD = 0.070 (39 to 39 atoms)

Figure S29. Molecular docking pose of 3-mercaptopyruvate (3-MP).

A) Sticks representation of 3-MP with enzyme *Ld*3MST in solid surface representation. B) Key interacting amino acid residues of *Ld*3MST with 3-MP. Only polar hydrogens are shown for clarity, H-bonds interactions are depicted as dash yellow lines at distances lower than 3.0 Å. 3-MP shows better pose and proximity to the catalytic Cys-253, compared to the co-crystalized sulfite ion inhibitor.

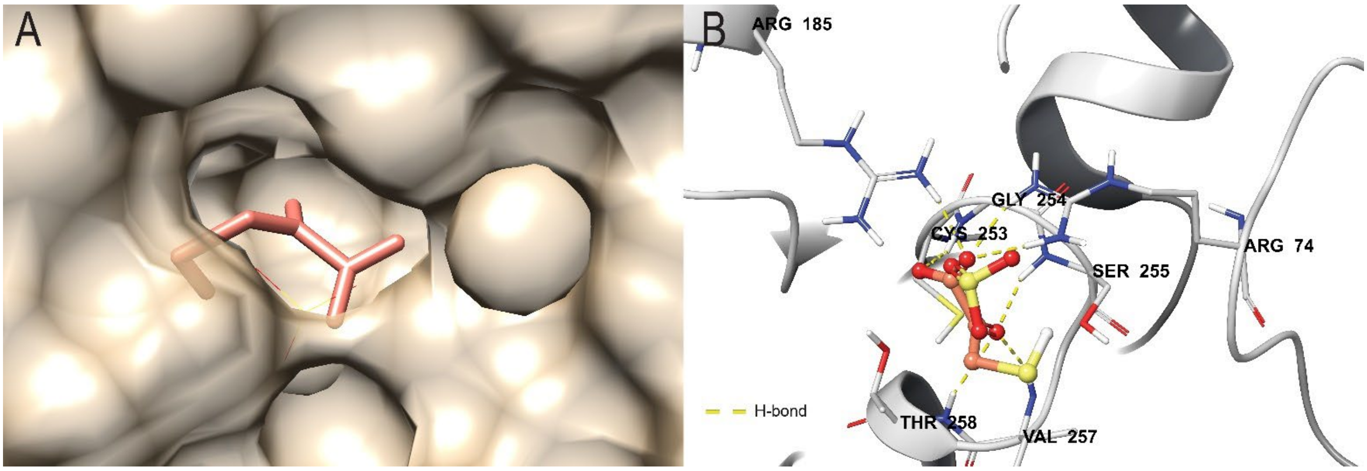

Figure S30. Preliminary molecular docking simulations performed with the active compounds and commonly used targets from *T. cruzi* and *Leishmania*.

A) Cruzipain from *T. cruzi* in complex with the hydroxymethyl ketone [1-(1-Benzyl-3-hydroxy-2-oxo-propylcarbamoyl)-2-phenyl-ethyl]-carbamic acid benzyl ester (PDB 1ME4). B) *T. cruzi* pteridine reductase 2 (PTR2) in complex with inhibitor methotrexate and the NADPH cofactor (PDB 1MXF). C) *T. cruzi* trans-sialidase in complex with substrate analog 3-deoxy-2,3-dehydro-*N*-acetylneuraminic acid (PDB 1MS1). D) *L. donovani* ornithine decarboxylase in complex with the pyridoxal phosphate (PLP) cofactor and inhibitor 3-aminooxy-1-aminopropane (APA) (PDB 2OO0). E) *L. major* 3-mercaptopyruvate sulfurtransferase (3MST) in complex with substrate mercaptopyruvate and a sulfite ion (PDB 1OKG). F) *L. donovani* pteridine reductase 1 (PTR1) in complex with inhibitor methotrexate and the NADPH cofactor (PDB 1E7W). In all figures, our compounds are colored in red, while the co-crystallized substrates, cofactors, and inhibitors are colored in green, blue, and orange, respectively. Docking binding scores calculated according to the protocol described in the article are shown below each compound name.

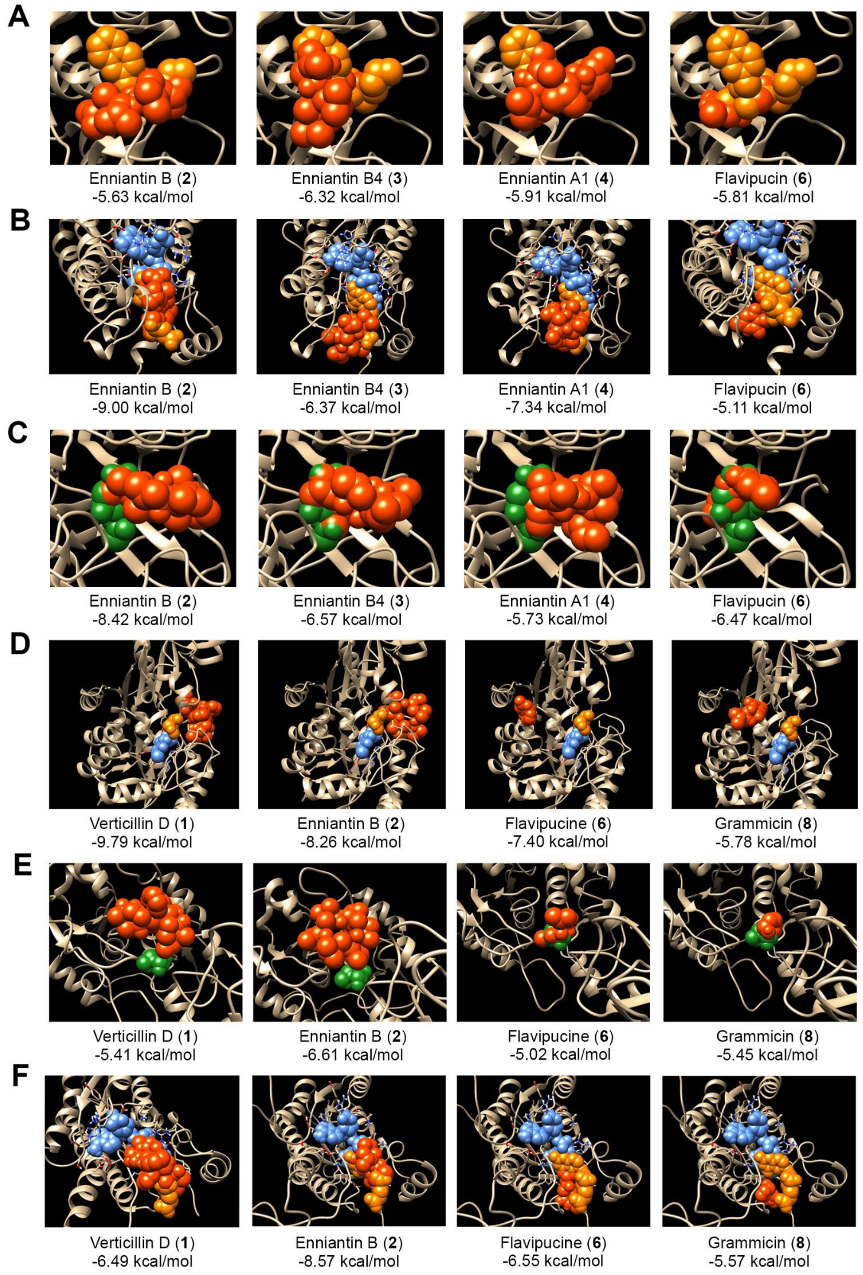

Reference

1. Joshi, B.K.; Gloer, J.B.; Wicklow, D.T. New Verticillin and Glisoprenin Analogues from *Gliocladium Catenulatum* , a Mycoparasite of *Aspergillus Flavus* Sclerotia. *J Nat Prod* 1999, *62*, 730–733, doi:10.1021/np980530x.
2. Tsantrizos, Y.S.; Xu, X.-J.; Sauriol, F.; Hynes, R.C. Novel Quinazolinones and Enniatins from *Fusarium Lateritium* Nees. *Can J Chem* 1993, *71*, 1362–1367, doi:10.1139/v93-176.
3. Tomoda, H.; Nishida, H.; Huang, X.-H.; Masuma, R.; Kim, Y.K.; Omura, S. New Cyclodepsipeptides, Enniatins D, E and F Produced by *Fusarium* Sp. FO-1305. *J Antibiot (Tokyo)* 1992, *45*, 1207–1215, doi:10.7164/antibiotics.45.1207.
4. Visconti, A.; Blais, L.A.; ApSimon, J.W.; Greenhalgh, R.; Miller, J.D. Production of Enniatins by *Fusarium Acuminatum* and *Fusarium Compactum* in Liquid Culture: Isolation and Characterization of Three New Enniatins, B2, B3, and B4. *J Agric Food Chem* 1992, *40*, 1076–1082, doi:10.1021/jf00018a034.
5. Li, Y.; He, N.; Luo, M.; Hong, B.; Xie, Y. Application of Untargeted Tandem Mass Spectrometry with Molecular Networking for Detection of Enniatins and Beauvericins from Complex Samples. *J Chromatogr A* 2020, *1634*, 461626, doi:10.1016/j.chroma.2020.461626.
6. Renaud, J.B.; Kelman, M.J.; McMullin, D.R.; Yeung, K.K.-C.; Sumarah, M.W. Application of C8 Liquid Chromatography-Tandem Mass Spectrometry for the Analysis of Enniatins and Bassianolides. *J Chromatogr A* 2017, *1508*, 65–72, doi:10.1016/j.chroma.2017.05.070.
7. Zaher, A.M.; Makboul, M.A.; Moharram, A.M.; Tekwani, B.L.; Calderón, A.I. A New Enniatin Antibiotic from the Endophyte *Fusarium Tricinctum* Corda. *J Antibiot (Tokyo)* 2015, *68*, 197–200, doi:10.1038/ja.2014.129.
8. Asha, K.N.; Chowdhury, R.; Hasan, C.M.; Rashid, M.A. Steroids and Polyketides from *Uvaria Hamiltonii* Stem Bark. *Acta Pharm* 2004, *54*, 57–63.
9. Loesgen, S.; Bruhn, T.; Meindl, K.; Dix, I.; Schulz, B.; Zeeck, A.; Bringmann, G. (+)-Flavipucine, the Missing Member of the Pyridione Epoxide Family of Fungal Antibiotics. *European J Org Chem* 2011, *2011*, 5156–5162, doi:10.1002/ejoc.201100284.
10. Edwards, R.L.; Maitland, D.J.; Pittayakhajonwut, P.; Whalley, A.J.S. Metabolites of the Higher Fungi. Part 33. Grammicin, a Novel Bicyclic C7H6O4 Furanopyranol from the Fungus *Xylaria Grammica* (Mont.) Fr. *J Chem Soc Perkin 1* 2001, 1296–1299, doi:10.1039/b101708j.
